# Drug-Resistant Epilepsy: Multiple Hypotheses, Few Answers

**DOI:** 10.3389/fneur.2017.00301

**Published:** 2017-07-06

**Authors:** Fei Tang, Anika M. S. Hartz, Björn Bauer

**Affiliations:** ^1^Department of Pharmacy Practice and Pharmaceutical Sciences, College of Pharmacy, University of Minnesota, Duluth, MN, United States; ^2^Department of Pharmaceutical Sciences, College of Pharmacy, University of Kentucky, Lexington, KY, United States; ^3^Sanders-Brown Center on Aging, University of Kentucky, Lexington, KY, United States; ^4^Department of Pharmacology and Nutritional Sciences, College of Medicine, University of Kentucky, Lexington, KY, United States; ^5^Epilepsy Center, University of Kentucky, Lexington, KY, United States

**Keywords:** epilepsy, refractory epilepsy, blood–brain barrier, P-glycoprotein, transporter hypothesis, target hypothesis, transporter inhibition, transporter regulation

## Abstract

Epilepsy is a common neurological disorder that affects over 70 million people worldwide. Despite the recent introduction of new antiseizure drugs (ASDs), about one-third of patients with epilepsy have seizures refractory to pharmacotherapy. Early identification of patients who will become refractory to ASDs could help direct such patients to appropriate non-pharmacological treatment, but the complexity in the temporal patterns of epilepsy could make such identification difficult. The target hypothesis and transporter hypothesis are the most cited theories trying to explain refractory epilepsy, but neither theory alone fully explains the neurobiological basis of pharmacoresistance. This review summarizes evidence for and against several major theories, including the pharmacokinetic hypothesis, neural network hypothesis, intrinsic severity hypothesis, gene variant hypothesis, target hypothesis, and transporter hypothesis. The discussion is mainly focused on the transporter hypothesis, where clinical and experimental data are discussed on multidrug transporter overexpression, substrate profiles of ASDs, mechanism of transporter upregulation, polymorphisms of transporters, and the use of transporter inhibitors. Finally, future perspectives are presented for the improvement of current hypotheses and the development of treatment strategies as guided by the current understanding of refractory epilepsy.

## Background: Refractory Epilepsy

Epilepsy is a common and devastating neurological disorder, affecting more than 70 million people worldwide (1). Epilepsy patients have recurrent unprovoked seizures, which can be focal or generalized in nature (2, 3). As a first line of treatment, antiseizure drugs (ASDs) are routinely used to control seizures. However, about one-third of epilepsy patients suffer from uncontrolled seizures despite pharmacotherapy (4). Although a unifying and precise definition of “refractory epilepsy” is not available (5), an epilepsy is generally considered “refractory,” “drug-resistant,” or “intractable” when seizures cannot be controlled by at least two or three ASDs appropriate for the particular epilepsy type (6–9). In this regard, the International League Against Epilepsy (ILAE) Task Force proposed that “[d]rug-resistant epilepsy may be defined as failure of adequate trials of two tolerated and appropriately chosen and used ASD schedules (whether as monotherapies or in combination) to achieve sustained seizure freedom” (10).

**Figure 1:**
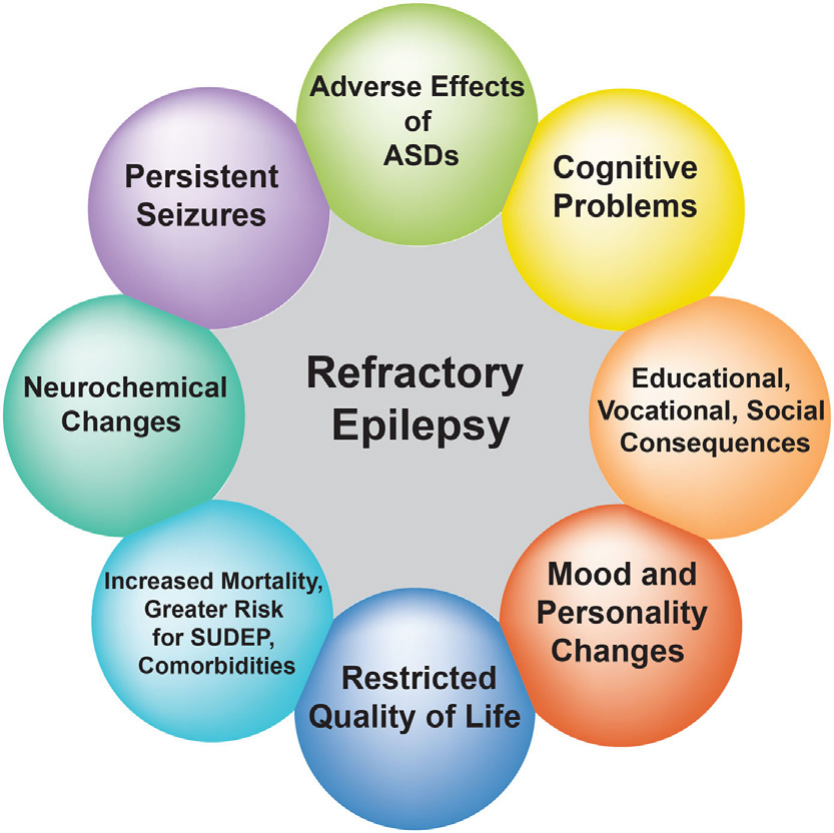
Effect of refractory epilepsy on patients’ quality of life. The circles depict the impact of recurrent seizures on the quality of life of patients with refractory epilepsy.

Refractory epilepsy is associated with increased morbidity and mortality, serious psychosocial consequences, cognitive problems, and reduced quality of life (Figure 1) (11–13). Despite the introduction of many new ASDs since 1990s, there has been little improvement in the prognosis of common epilepsies and childhood epilepsy syndromes (14, 15). This is not surprising given the lack of compelling evidence supporting the superiority of new ASDs over older ones, as well as the small placebo-corrected efficacy of adjunctive treatment with modern ASDs (16, 17).

As part of this background section on refractory epilepsy, we will briefly cover management, temporal patterns, and predictors of refractory epilepsy, and then discuss the existing hypotheses that have been proposed to explain the potential mechanisms underlying ASD resistance.

### Management of Refractory Epilepsy

**Figure 2:**
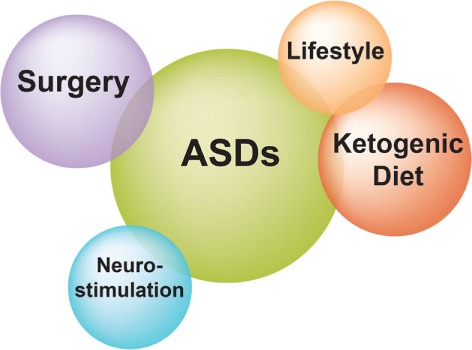
Treatment strategies for refractory epilepsy. Current treatment options for patients with refractory epilepsy include pharmacotherapy with antiseizure drugs, surgical removal of the seizure focus, and alternative approaches such as neurostimulation, ketogenic diet, and lifestyle changes.

Patients with refractory epilepsy carry the greatest burden of treatment of epilepsy (18). Management strategies of refractory epilepsy fall into three main categories: pharmacotherapy, epilepsy surgery, and alternative treatment strategies including neurostimulation, ketogenic diet, and lifestyle changes (Figure 2) (18). With regard to pharmacotherapy, clinical evidence shows that patients who do not respond to two ASDs have only a small chance to control their seizures with any additional administered ASD (10). In a recently published prospective cohort study with 1,098 newly diagnosed epilepsy patients who were recruited between 1982 and 2006 and were followed for up to 26 years (until 2008), Brodie et al. (19) found that 49.5% of enrolled patients remained seizure-free (i.e., not experiencing seizures for at least 1 year) on their first ASD, while only 13.3, 3.7, 1.0, and 0.4% of the cohort became seizure-free on the second, third, fourth, and fifth regimen (either as monotherapy or in combination), respectively (Table 1). However, since a few patients did achieve sustained seizure freedom while on the fourth up to the seventh medication regimen, patients who failed the first three ASD regimens did not inevitably become refractory (19).

**Table 1:** Success rates of achieving seizure freedom with successive antiseizure drug (ASD) regimens.

Number of ASDs	Number of patients	Number of seizure-free patients	Seizure-free patients (% of total cohort)
1	1,098	543	49.5
2	398	146	13.3
3	168	41	3.7
4	68	11	1.0
5	32	4	0.4
6	16	2	0.2
7	9	2	0.2
8	3	0	0
9	2	0	0

*The chance of seizure freedom declines with successive ASD regimens, most markedly from the first to the third, among patients with epilepsy. Modified from Brodie et al. (19)*.

In patients with refractory epilepsy who do not respond to ASDs, other therapeutic avenues are pursued including surgery (18). In this regard, patients with refractory epilepsy caused by distinct resectable lesions, such as hippocampal sclerosis (HS), are potential candidates for neurosurgical removal of the lesion (20). Epilepsy surgery has been shown to be superior to the continued use of ASDs, but the supporting clinical evidence from randomized controlled trials is limited to temporal lobe epilepsy (TLE) (21).

An alternative treatment approach is neurostimulation such as vagus nerve stimulation and responsive neurostimulation (22). Vagus nerve stimulation can reduce the frequency and/or severity of seizures (20), but some patients experience adverse effects such as hoarseness, coughing, and dyspnea (23). Responsive neurostimulation is a novel treatment that was approved in the US in 2013 for adults with focal onset epilepsy (23). Neurostimulation is an invasive, intracranial procedure, and its efficacy does not significantly differ from other neurostimulation treatments (22).

Another option is to switch to a ketogenic diet, an approach that is more commonly used in children with refractory epilepsy and, while the underlying mechanism remains unknown, has demonstrated high efficacy rates with some studies showing that about half the patients had a more than 50% reduction in seizure numbers (23). However, ketogenic diet is challenging for children due to compliance difficulties and potential short-term and long-term adverse effects and, therefore, requires regular follow-up and clinical supervision (24).

Finally, certain lifestyle changes can help to control seizures by minimizing seizure triggers. Common seizure triggers include sleep deprivation, interrupted sleep, longer periods without food, alcohol, caffeine, nicotine, drugs of abuse, psychological stress, emotional tension, and sensory input (e.g., photosensitivity, strobe light, and computer and video games). Therefore, sufficient sleep, managing stress levels effectively, and following a healthy lifestyle can help with seizure control to some extent.

In summary, pharmacotherapy is the mainstay of epilepsy management. Epilepsy surgery and alternative measures including neurostimulation and ketogenic diet are among the therapeutic options for patients with refractory epilepsy, each with its own advantages and disadvantages. While seizures cannot fully be prevented by lifestyle changes alone, these changes can contribute to improving quality of life and helping with seizure control. For treatment purposes, each patient’s unique circumstances need to be taken into consideration when selecting the appropriate management strategy (18).

### Temporal Patterns of Refractory Epilepsy

Pharmacoresistance in epilepsy was thought to be constitutive or progressive, and consequently, one clinical paradigm postulated that an early response to ASD therapy indicates a favorable prognosis (25). However, accumulating evidence now demonstrates a higher level of complexity of the temporal patterns of epilepsy, i.e., the clinical courses and response patterns to ASDs (15). In the cohort study of Brodie et al. (19), 37% (408 patients) of a total of 1,098 epilepsy patients achieved sustained seizure freedom within 6 months of initiating ASD therapy, 22% of patients achieved sustained seizure freedom that was delayed for over 6 months after treatment initiation, 16% of patients fluctuated between seizure freedom and relapse, and 25% of patients never achieved seizure freedom for at least 1 year. Of the 408 patients who followed the first temporal pattern, the majority became seizure-free on the first monotherapy regimen, 37 required a second regimen (either an alternative monotherapy or combination regimen), and 4 required a third regimen (19). Callaghan et al. (26) conducted a prospective cohort study with 246 ASD-resistant patients and found that on average 5% of patients per year gained seizure freedom for at least 1 year over 6 years of follow-up, but the risk of relapse among those patients was relatively high with 71% after 5 years. The authors of the study also noted that the remission was negatively correlated with the number of ASDs that failed in a particular patient, while relapse could not be explained by dose reductions or medication discontinuation alone. Similarly, Neligan et al. (27) conducted a prospective cohort study in 139 patients with uncontrolled chronic epilepsy with a median follow-up of 6.9 years and showed that 19% of patients became seizure free and 29% of patients experienced 50–99% improvement in seizure frequency at the last follow-up. However, a substantial proportion of the patients who experienced remission subsequently relapsed (27). In another study, Neligan et al. (28) found that the intermittent pattern of seizures (i.e., having one or more seizure-free periods which lasted for at least 2 years) occurred in about 30% of patients with refractory epilepsy, which was found to be associated with fewer total ASDs taken and lower seizure frequency in the previous year when compared to the continuous pattern of pharmacoresistance.

In summary, recent studies have shown that the temporal patterns of refractoriness in epilepsy are more complex than previously assumed, and up to 30% of patients with refractory epilepsy follow a fluctuating course with periods of remission and relapse. Based on these observations, achievement of sustained seizure freedom may be a result of both the development course of benign epilepsy and the treatment effect of ASDs, but it is unclear at this point how much each of the two contributes to long-term remission (28).

### Predictors of ASD Resistance

Some have suggested that early identification of epilepsy patients who will become refractory to ASDs could help directing these patients to appropriate non-pharmacological treatment (3, 9, 29). However, others have argued that such identification can be difficult given that a considerable number of patients may have alternating periods of relapse and remission (22). Nevertheless, outcome studies in epilepsy have identified several factors that have repeatedly been shown to be predictive of a poor prognosis, including the initial response to pharmacotherapy, the underlying etiology, and a patient’s history of seizure frequency (3). Specifically, inadequate response to initial ASD therapy has been shown to be the most powerful indicator of refractory epilepsy (3, 4). Symptomatic epilepsy characterized by structural brain abnormality tends to be more ASD-resistant than idiopathic epilepsy, which presumably has an underlying genetic basis (13, 29). A high frequency of pretreatment seizures has also been found to be a poor prognostic factor. On the other hand, factors such as seizure types and electroencephalogram findings did not consistently show significant prognostic value (3).

Together, prognostic factors are useful in predicting refractoriness in some but not all patient cases (29), and more importantly, none of these factors explains the underlying mechanism of pharmacoresistance (6, 11).

## Potential Mechanisms of ASD Resistance

**Figure 3:**
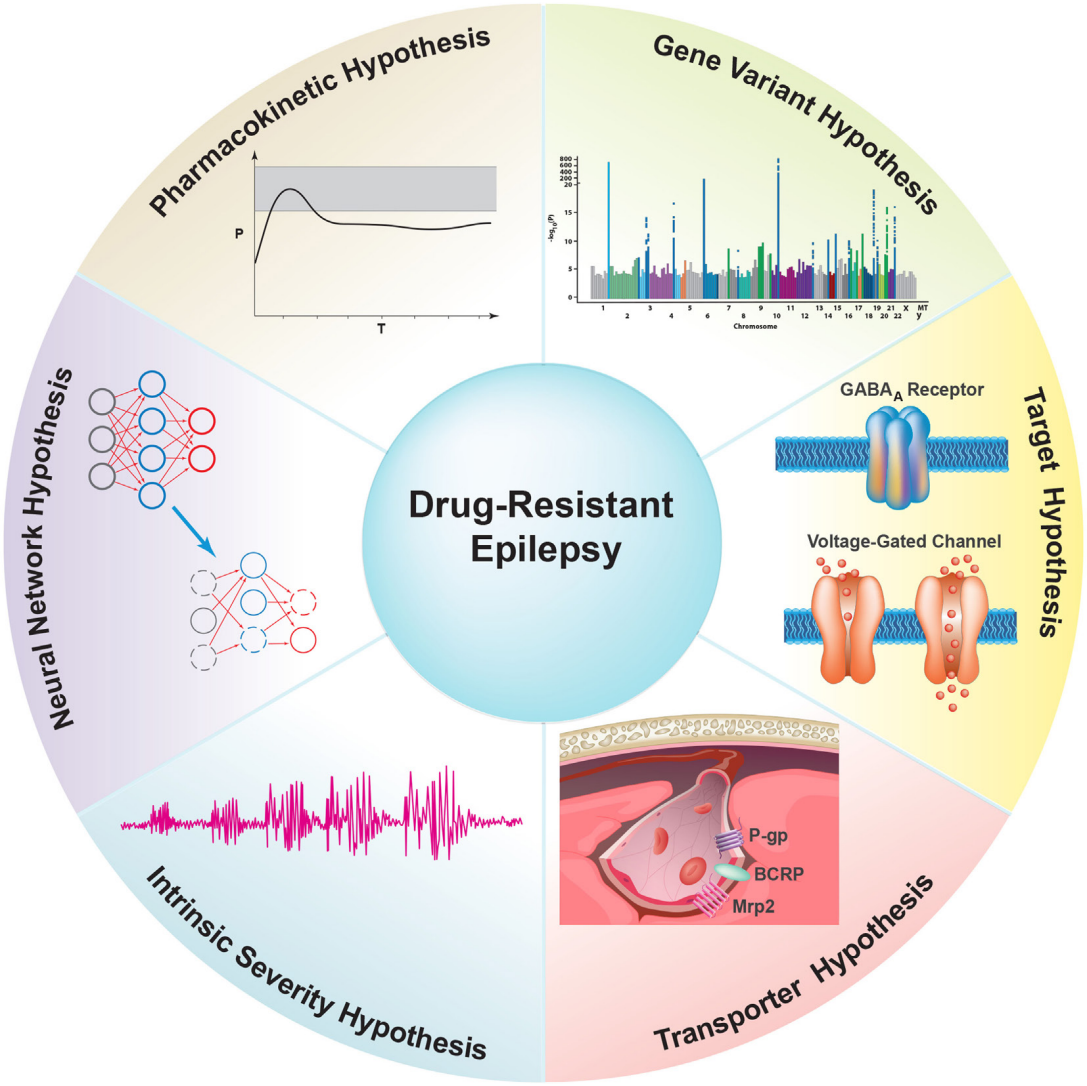
Overview of proposed hypotheses for possible underlying mechanism(s) of antiseizure drug (ASD) resistance. (1) The *Pharmacokinetic Hypothesis* proposes that overexpression of drug efflux transporters in peripheral organs decreases ASD plasma levels, thereby reducing the amount of ASD available to enter the brain and reach the epileptic focus. (2) The *Neuronal Network Hypothesis* states that seizure-induced degeneration and remodeling of the neural network suppresses the brain’s seizure control system and restricts ASDs from accessing neuronal targets. (3) The *Intrinsic Severity Hypothesis* proposes that common neurobiological factors contribute to both epilepsy severity and pharmacoresistance (30). (4) The *Gene Variant Hypothesis* states that variations in genes associated with ASD pharmacokinetics and pharmacodynamics cause inherent pharmacoresistance. These genes include metabolic enzymes, ion channels, and certain neurotransmitter receptors that are targets for ASDs. (5) The *Target Hypothesis* postulates that alterations in the properties of ASD targets, such as changes in voltage-gated ion channels and neurotransmitter receptors (e.g., GABA_A_ receptor), result in decreased drug sensitivity and thus lead to refractoriness. (6) The *Transporter Hypothesis* states that overexpression of ASD efflux transporters at the blood–brain barrier in epilepsy leads to decreased ASD brain uptake and thus ASD resistance.

Understanding the mechanism(s) underlying ASD resistance has the potential to help the development of more effective therapeutic options for patients with refractory epilepsy. The target hypothesis and transporter hypothesis are the most cited theories of ASD resistance, but neither fully explains the neurobiological basis of this phenomenon (30, 31). It is clear that the mechanism(s) of refractory epilepsy is/are most likely multifactorial, involving environmental, genetic, as well as disease- and drug-related factors (32, 33). In the following sections, we discuss several hypotheses that have been proposed, starting with the least cited to the most cited: (1) the pharmacokinetic hypothesis, (2) the neural network hypothesis, (3) the intrinsic severity hypothesis, (4) the gene variant hypothesis, (5) the target hypothesis, and finally the (6) transporter hypothesis, which will be the main focus of this review (Figure 3).

### Pharmacokinetic Hypothesis

The pharmacokinetic hypothesis proposes that overexpression of efflux transporters in peripheral organs such as intestine, liver, and kidney decreases ASD plasma levels in refractory epilepsy patients, thereby reducing the amount of ASD available to cross the blood–brain barrier and reach the epileptic focus in the brain (34).

In a case report of a pediatric patient with refractory epilepsy, Lazarowski et al. (35) detected persistently low plasma levels of carbamazepine, phenytoin, and valproic acid. This coincided with increased P-glycoprotein (P-gp) protein expression levels in endothelial cells, astrocytes, and neurons from the patient’s resected brain tissue. In another case report of a pediatric patient with refractory epilepsy, the same group described persistently low phenytoin plasma levels and increased P-gp protein expression analyzed by immunohistochemistry in resected epileptic brain tissue (36). The authors also reported that the P-gp substrate, ^99m^Tc-hexakis-2-methoxyisobutylisonitrile, demonstrated increased hepatic clearance in eight patients with refractory epilepsy compared to seven normal subjects and four patients with controlled epilepsy (37). Based on this finding, the authors postulated that the liver is involved in potential pharmacokinetic changes that could contribute to ASD resistance (34).

In the two cases described above, the authors argued that subtherapeutic ASD blood levels could not be explained by overexpression of P-gp at the blood–brain barrier and in neurons. Instead, the authors suggested overexpression of P-gp or other efflux transporters in the periphery as an additional mechanism for refractory epilepsy, especially in patients who presented with persistently low ASD plasma levels (34). While this explanation is plausible, the authors postulated their hypothesis based on only two case studies, and it is unclear at this point if their observation is limited to these cases or a wider-spread phenomenon. In addition, the authors did not provide any additional evidence from human samples and/or from rodent epilepsy models to substantiate their statements.

Support for the pharmacokinetic hypothesis also comes from studies showing persistent low ASDs levels in patients with refractory epilepsy regardless of P-gp overexpression. For example, in a clinical study of 70 patients treated with oral phenytoin, Iwamoto et al. (38) found that the mean free phenytoin plasma concentration was significantly higher in patients with a complete response to phenytoin compared to patients with a partial response. This effect was independent of the phenytoin dose, and the results suggest that the free phenytoin concentration could be useful for monitoring ASDs effects in patients receiving phenytoin monotherapy. In a retrospective study, Paul and coworkers (39) found in 80% of patients with refractory epilepsy that lamotrigine serum levels were decreased by 20% after surgery compared to preoperative levels. In six patients, seizures were observed within the first 2 weeks after surgery. In three of these patients, seizures occurred after reaching the nadir of lamotrigine plasma levels. Therefore, the authors propose counteracting a postoperative reduction in serum lamotrigine levels by augmenting the preoperative drug dose and close monitoring of drug serum levels after surgery (39). Dalaklioglu (40) reported a high frequency of subtherapeutic ASD plasma levels in patients with refractory epilepsy. Further, Fagiolino et al. (41) conducted a clinical study and observed that the saliva drug concentration ratio from two sequentially collected samples could be utilized to detect systemic clearance changes. This could be useful to predict plasma levels of ASDs such as carbamazepine and phenytoin that are known to induce drug efflux transporters during chronic treatment.

Other studies suggest an association between peripheral expression levels of metabolizing enzymes and efflux transporters on the one hand and plasma ASD concentrations on the other hand. Kerb et al. (42) reported a clinical study conducted in 96 healthy Turkish volunteers. In this study, the combined analysis of CYP2C9 and multidrug resistance protein 1 (MDR1) genotypes had better predictive value for phenytoin plasma concentrations than CYP2C9 analysis alone. Simon et al. (43) found that increased intestinal P-gp expression levels had a weak association with low carbamazepine plasma concentrations, and increased intestinal MRP2 expression levels were weakly related to high carbamazepine doses in 29 epilepsy patients. Nevertheless, unlike the case reports by Lazarowski et al., neither study directly addressed ASD response.

In addition, data from clinical studies show that ASD-responsive and ASD-resistant patients display adverse events to the same extent (44, 45), suggesting similar plasma ASD levels in the two groups of patients. One explanation for this observation is that efflux transporter overexpression is restricted to the epileptic focus. This observation also suggests that same plasma ASD concentrations are due to same enzyme and transporter expression levels in peripheral organs. While both of these explanations are plausible, one does not necessarily lead to the other.

Furthermore, some animal studies do not support the pharmacokinetic hypothesis. In these studies, differences in ASD plasma concentrations and/or side effects have not been observed between ASD responders and non-responders (46, 47), and administration of transporter inhibitors enhanced anticonvulsant activity of the ASD without changing its pharmacokinetics (44, 47, 48).

Together, the pharmacokinetic hypothesis of refractory epilepsy as a stand-alone theory is difficult to validate. One can argue that because abnormalities in ASD plasma concentrations can be readily captured by therapeutic drug monitoring, pharmacokinetic variability is probably not a major contributor to pharmacoresistance in situations where ASD doses are adjusted accordingly. This argument, however, is further complicated because therapeutic ASD plasma concentrations vary among patients, and no one specific therapeutic ASD concentration range is applicable to all patients (49, 50). The optimal plasma concentration for a patient is partially related to the patient’s seizure type and disease severity, as well as the pharmacodynamic characteristics of the specific ASD(s) used (49, 50). For newer ASDs, wide ranges of therapeutic serum concentrations have also been reported, and concentrations corresponding to toxicity and non-response can overlap considerably (50). Therefore, it seems more appropriate to adjust ASD dosages on an individual basis than to strictly conform to reference therapeutic plasma concentrations (49).

### Neural Network Hypothesis

Recently, Fang et al. (51) proposed the neural network hypothesis, which states that seizure-induced degeneration and remodeling of the neural network suppress the endogenous antiseizure system and inhibit ASDs from accessing neuronal targets. Specifically, molecular evidence shows that the growth cone at the tip of an exon receives abnormally expressed guidance and signaling molecules in the epileptic brain (51). In addition, the formation of new excitatory circuits as a result of progressive sprouting has been widely investigated in TLE (51). The authors postulate that neurogenesis and astrogliosis in TLE could contribute to the development of abnormal neural networks and eventually ASD resistance. However, the major weakness of this hypothesis is that alterations in the neural network do not lead to refractoriness in all epilepsy patients, and therefore, further biological evidence on potential differences in the changes of brain plasticity between drug-responsive and drug-resistant epilepsy is needed to support this hypothesis (51).

### Intrinsic Severity Hypothesis

The intrinsic severity hypothesis states that common neurobiological factors contribute to both epilepsy severity and pharmacoresistance (30). In other words, pharmacoresistance is inherent to the disease severity, which could exist on a continuum ranging from mild to severe (52).

In this regard, data from reports supporting the intrinsic severity hypothesis suggest that high pretreatment seizure frequency is an important predictor for refractory epilepsy (53–55). Based on these reports, it is tempting to draw an association between ASD resistance and the experimental electrical kindling, in which repeated electrical stimulation at a subconvulsive level can eventually induce spontaneous recurrent seizures in animals (4, 13). However, a randomized clinical study demonstrated that starting ASD treatment after the first tonic–clonic seizure did not improve the prognosis of epilepsy (56). In fact, the same probability of becoming seizure-free for 1 or 2 years was seen in patients who were treated after the first seizure and those who received treatment after seizure recurrence (56). A similar conclusion was drawn from a cohort study in children with epilepsy, which showed that ASD administration at some point during the first 10 seizures had no aggravating effect on achieving seizure control or early remission (57). In a randomized study in 1,847 epilepsy patients, the authors compared immediate and deferred treatment with ASDs and found that immediate treatment was associated with seizure reduction in the first 1–2 years, but rates of long-term remission did not differ between the two groups (58).

Therefore, such findings argue against the notion of a kindling-like process where the likelihood of ASD resistance is increased with the number of pretreatment seizures. Instead, high seizure frequency prior to ASD treatment could be the result of pathophysiological changes characterizing refractory epilepsy (4). An alternative interpretation of the epidemiological data resulted in the intrinsic severity hypothesis. While this theory appears biologically plausible, it does not adequately apply to epilepsy types that demonstrate a fluctuating or evolving pattern of ASD resistance (30). In addition, there is little evidence supporting a direct mechanistic link between the severity of epilepsy and ASD response (59). Therefore, it has been suggested that the intrinsic severity theory alone does not sufficiently explain pharmacoresistance in epilepsy (59).

### Gene Variant Hypothesis

The gene variant hypothesis states that variations in genes associated with ASD pharmacokinetics and pharmacodynamics cause inherent pharmacoresistance (17). Specifically, variations in genes that encode enzymes that metabolize ASDs or ion channels and neurotransmitter receptors targeted by ASDs can potentially affect ASD response (33).

Phenytoin is metabolized by CYP2C9 (90%) and CYP2C19 (60). Van der Weide et al. (61) reported strong associations between the low activity alleles of *CYP2C9* (CYP2C9*2 and CYP2C9*3) and a reduced phenytoin dose requirement. In a different study, Tate et al. (60) also revealed a significant correlation between CYP2C9*3 and a reduced dose requirement of phenytoin, and Ufer et al. (62) found in a specific subgroup of patients significantly more heterozygous CYP2C8*4 and CYP2C9*3 variant allele carriers among ASD responders compared to ASD non-responders.

Voltage-gated sodium channels are the target of several commonly used ASDs, including carbamazepine, phenytoin, lamotrigine, and valproate (63). Voltage-gated sodium channels consist of one α subunit and two β subunits. The isoforms of the α subunits, Na_v_1.1, 1.2, 1.3, and 1.8, are encoded by the *SCN1A, 2A, 3A*, and *8A* genes, respectively (64). Using a haplotype-tagging strategy, Tate et al. (60) demonstrated a significant correlation between an intronic single nucleotide polymorphism (SNP) in the *SCN1A* gene (IVS5-91G>A or rs3812718) and the maximum required doses of carbamazepine and phenytoin in groups of 425 and 281 English patients, respectively. In a follow-up study in 168 Chinese epilepsy patients on phenytoin treatment, Tate et al. (65) found that the same polymorphism was correlated with phenytoin serum levels at maintenance dose, but not with the maintenance or maximum dose of phenytoin. In a study including 228 Japanese patients with epilepsy, Abe et al. (66) demonstrated a significant association between the frequency of the *SCN1A* IVS5-91 AA genotype and resistance to carbamazepine, but not the carbamazepine maximum or maintenance dose. Kwan et al. (64) genotyped tagging and candidate SNPs of *SCN1A, 2A*, and *3A* in 471 Chinese patients with epilepsy and reported a significant correlation between an intronic SNP in *SCN2A* (IVS7-32A>G, rs2304016) and responsiveness to various ASDs, but the polymorphism did not significantly alter *SCN2A* mRNA levels in resected brain tissue or peripheral white blood cells. On the other hand, the association between IVS5-91G>A in the *SCN1A* gene and ASD response was not observed in this study (64). Several more recent studies explored the relationship between other SNPs in the sodium channel genes and drug response in epilepsy, including *SCN1A* c.3184 A>G (rs2298771) and *SCN2A* c.56 G>A (rs17183814), both of which were found to be functionally significant in some neurological disorders (67–69). In a study including 336 epilepsy patients from the northern part of India, Lakhan et al. (67) reported a significant association between the variant allele frequency of *SCN2A* c.56 G>A SNP and ASD resistance. This finding was confirmed by Kumari et al. (68) in another study with 402 epilepsy patients from the same geographic region. Although Lakhan et al. and Kumari et al. did not reveal an association between *SCN1A* c.3184 A>G SNP and ASD resistance, Abo El Fotoh et al. (69) demonstrated a significant relationship between the AG genotype or G allele and ASD resistance in Egyptian children with epilepsy.

In summary, with the gene variant hypothesis, currently the strongest evidence exists for the association between *CYP2C9* polymorphism and phenytoin dose requirement. Although the relationship between various *SCN1A* and *SCN2A* polymorphisms and ASD dose requirement and/or response has been explored in a number of genetic association studies, the study results have been inconsistent, and genetic associations identified so far need further confirmation in larger populations. In addition, given the low frequency of certain alleles and the multifactorial nature of refractory epilepsy, it is possible that individual markers may not have a large enough clinical impact on overall ASD response (64). Together, the impact of the gene variant hypothesis as a stand-alone theory is mainly limited by inconsistencies and poor reproducibility of study findings. Nevertheless, improvement in genomic technologies and research methodology is expected to increase the chances of uncovering truly predictive genetic markers for ASD resistance and further the advancement of epilepsy pharmacogenomics (70).

### Target Hypothesis

The target hypothesis of refractory epilepsy postulates that alterations in the properties of ASD targets, such as compositional changes in voltage-gated ion channels and neurotransmitter receptors, result in decreased drug sensitivity and thus lead to refractoriness (63, 71). For example, loss of use-dependent blockade of voltage-gated sodium channels in dentate granule cells by carbamazepine was observed in rats after pilocarpine-induced epilepsy and in resected hippocampal tissue from patients with carbamazepine-resistant TLE (71). However, this loss in efficacy due to a potential change in the molecular target has so far only been reported for carbamazepine and has not been demonstrated to occur with other ASDs that block sodium channels (72). Reduced sensitivity of GABA_A_ receptors to agents that bind to the benzodiazepine receptor site 1 has been reported in the pilocarpine model of epilepsy (63), and data from two other studies showed changes in GABA_A_ receptor subtypes in brain tissue from patients with refractory TLE (73, 74). Overall, evidence supporting the target hypothesis mainly describes the loss of use-dependent channel blockade by carbamazepine and comes from resected human brain tissue (72, 75). The fact that most refractory patients are resistant to several ASDs acting on different therapeutic targets undermines the general utility of the target hypothesis and instead supports the existence of a mechanism non-specific to individual ASDs (2).

### Transporter Hypothesis

Multidrug resistance due to efflux transporters has been studied extensively in tumor cells. The best understood efflux transporters are members of the ABC (ATP-binding cassette) superfamily subfamilies B, C, and G, specifically P-gp (*ABCB1 or MDR1*), the multidrug resistance-associated proteins (MRP1, *ABCC1*; MRP2, *ABCC2*), and breast cancer resistance protein (BCRP, *ABCG2*) (75). Members of the ABC superfamily are ATP-driven membrane pumps that actively transport substrates, including a large number of therapeutic drugs, against their concentration gradient out of cells and tissues, limiting their entry into the respective organs and thereby causing resistance (75). For example, P-gp, BCRP, and some multidrug resistance-associated proteins (MRPs) hinder chemotherapeutic drugs from entering cancer cells. Thus, ABC transporter overexpression in cancer causes resistance to chemotherapeutic drugs resulting in poor prognosis in cancer patients (32, 76).

In 1995, Tishler et al. (77) found that *MDR1* mRNA was overexpressed in brain tissue resected from patients with refractory epilepsy and postulated the transporter hypothesis of refractory epilepsy: P-gp overexpression at the blood–brain barrier in epilepsy decreases ASD brain uptake, thus causing ASD resistance similar to pharmacoresistance in cancer (2). Since this initial proposal by Tishler et al., other ABC transporters have been shown to be upregulated at the blood–brain barrier in epilepsy and the transporter hypothesis has been intensively investigated (45). The transporter hypothesis is based on two assumptions: (1) overexpression of efflux transporters correlates with pharmacoresistance in epilepsy and (2) ASDs are subject to active transport by efflux transporters (78). In the following, we will describe the roles P-gp, the MRPs, and BCRP have in epilepsy in more detail.

#### P-Glycoprotein

P-glycoprotein is also known as MDR1 (old nomenclature) or ATP-binding cassette subfamily B member 1 (ABCB1, new nomenclature). P-gp is encoded by the *MDR1 (ABCB1)* gene in humans and by the *mdr1a/mdr1b* genes in rodents (79). P-gp protein is expressed in various barrier and excretory tissues such as intestine, liver, and kidney, where it actively exports hydrophobic and amphipathic molecules from the inside of cells or membranes to the outside (80, 81). This physiological function of exporting naturally occurring toxins and xenobiotics is considered to be a critical defense mechanism (82). In the normal human brain, P-gp is expressed in the luminal plasma membrane of the brain capillary endothelial cells that constitute the blood–brain barrier as well as in the apical membrane (facing the cerebrospinal fluid) of the choroid plexus epithelial cells that form the blood–cerebrospinal fluid barrier (83). P-gp expression is only marginally detectable in neurons or glial cells under normal, physiological conditions (32). In rodents, the *mdr1a* isoform is mainly expressed in endothelial cells of the blood–brain barrier, and *mdr1b* is primarily found in astrocytes (84).

#### Multidrug Resistance-Associated Proteins

The MRP family (ATP-binding cassette subfamily C, ABCC) comprises nine members (MRPs 1–9 or ABCCs 1–6 and 10–12) (85). MRPs are expressed in the membranes of various cell types, such as hepatocytes, kidney proximal tubular epithelial cells, enterocytes, and brain endothelial cells, where they transport a wide variety of mostly anionic endogenous and exogenous compounds and their metabolites (85). The luminal and/or basolateral localization of MRP proteins is often specific to a certain cell type (86). MRP1 is expressed at the basolateral membrane of choroid plexus epithelial cells and at low levels at the luminal membrane of endothelial cells at the blood–brain barrier (86). MRP2 is exclusively expressed at the luminal membrane of polarized cells, including brain endothelial cells (85). MRP4 and MRP5 have also been found to be apically localized in human brain capillary endothelial cells (87), whereas neuronal or glial MRP1 and MRP2 expression in the normal brain has not been consistently reported in the literature (32, 83, 88).

#### Breast Cancer Resistance Protein

Breast Cancer Resistance Protein (ATP-binding cassette subfamily G member 2 or ABCG2) is prominently expressed at the apical membrane in various cell types, including hepatocytes, intestinal epithelial cells, kidney proximal tubular cells, and the endothelial cells of the blood–brain barrier (76, 89). Similar to P-gp, BCRP transports a wide variety of substrates, and its tissue distribution contributes to its important roles in restricting absorption and facilitating elimination of drugs and xenobiotics (76).

##### Overexpression of Efflux Transporters in Refractory Epilepsy

P-glycoprotein overexpression in epileptogenic brain tissue in patients with refractory epilepsy has been documented in numerous studies (45). Tishler et al. (77) were the first to demonstrate overexpression of *MDR1* mRNA in 11 out of 19 resected brain specimens from patients with refractory focal epilepsy. Subsequently, increased levels of P-gp protein expression have also been observed in the brain capillary endothelium of resected brain tissue from patients with refractory epilepsy, where P-gp overexpression was localized to the luminal membrane of the brain capillary endothelium by immunohistochemistry (80, 90). P-gp overexpression was also detected in astrocytes and/or dysplastic neurons in common pathological causes of refractory epilepsy, including dysembryoplastic neuroepithelial tumors (DNT), HS, and focal cortical dysplasia (FCD) (32, 84, 88, 91–93).

MRP1 overexpression in astrocytes and/or dysplastic neurons in HS, DNT, and FCD has also been described in a number of studies (32, 88, 91, 93). The results from these studies confirm that MRP1 protein expression levels in astrocytes and neurons from brain tissue of epilepsy patients are significantly increased compared to brain tissue from healthy individuals, while endothelial MRP1 expression did not differ between the two (94).

Dombrowski et al. (80) were the first to report increased *MRP2* and *MRP5* mRNA levels in endothelial cells isolated from epileptic brain tissue of patients with refractory epilepsy compared to control endothelial cells from human umbilical vein and aneurysm domes. Aronica et al. (88) reported MRP2 protein overexpression in endothelial cells and astrocytes in HS tissue specimens of adult patients with TLE. The same observation was reported by Vogelgesang et al. (92) for MRP2 protein in DNT tissue from patients with refractory epilepsy. In the same study, the authors also observed MRP5 protein overexpression in dysplastic neurons, astrocytes, and brain endothelial cells in epileptogenic tissue.

Data from few studies comparing BCRP expression in control and epileptic human brain tissue demonstrated the constitutive expression of BCRP in the brain capillary endothelium, but these data do not show differences in BCRP expression levels between the groups (89, 90, 92, 95). Due to the current lack of evidence on BCRP overexpression in human epileptic brain tissue, BCRP is unlikely a major player in ASD resistance as proposed by the transporter hypothesis.

Although increased mRNA and protein expression levels of P-gp and MRPs have been demonstrated in resected brain tissue from patients with ASD-resistant epilepsy, previous studies did not include proper controls, as it is generally difficult to obtain brain tissue from either patients with drug-responsive epilepsy or from healthy subjects without brain disease. Therefore, it is still unclear if overexpression of efflux transporters correlates with and potentially causes ASD resistance, or if it is an epiphenomenon of epilepsy in humans that is unrelated to ASD resistance (96).

In this regard, Volk and Löscher established a correlation between ASD response and P-gp expression levels in a rat model of TLE with sustained spontaneous recurrent seizures developed after electrically induced status epilepticus (SE) (46). Using this model, the authors demonstrated that epileptic rats that did not respond to phenobarbital had higher P-gp expression levels in the capillary endothelial cells of the limbic brain region compared to rats that responded to phenobarbital (46). In humans, non-invasive positron emission tomography (PET) imaging is one approach to directly compare P-gp functional activity in ASD-responsive vs. ASD-resistant patients by determining tissue concentrations of PET tracers that are P-gp substrates (97). In a small pilot PET study using the P-gp substrate (R)-[^11^C]verapamil, Langer et al. (98) reported no significant differences in pharmacokinetic parameters between epileptogenic and non-epileptogenic brain regions in patients with refractory unilateral TLE. Subsequently, Feldmann et al. (99) conducted a PET study in 14 patients with ASD-refractory TLE, 8 patients with ASD-controlled TLE, and 13 healthy control individuals. In patients with refractory TLE, (R)-[^11^C]verapamil brain uptake was reduced compared to seizure-free patients, and the increase in (R)-[^11^C]verapamil brain uptake following the administration of tariquidar (P-gp inhibitor) was smaller compared to healthy individuals; both observations are consistent with higher P-gp activity at the blood–brain barrier in patients with refractory TLE (99). This study was the first to provide direct *in vivo* evidence of P-gp overactivity in patients with refractory epilepsy. In a more recent study of Shin et al. (100) in six patients with ASD-resistant epilepsy, five patients with ASD-responsive epilepsy, and eight healthy subjects, (R)-[^11^C]verapamil PET and magnetic resonance (MR) imaging with cyclosporine A (P-gp inhibitor) demonstrated significant asymmetry of P-gp expression in refractory patients compared to both seizure-free patients and healthy subjects, suggesting higher P-gp expression and lower uptake of (R)-[^11^C]verapamil in the group of patients with refractory epilepsy. Larger PET studies comparing transporter activity at the blood–brain barrier in ASD-responsive and ASD-resistant patients are needed in the future to confirm the results presented above.

In summary, overexpression of ABC multidrug efflux transporters at the blood–brain barrier observed in numerous studies forms the foundation of the transporter hypothesis of refractory epilepsy. In addition, astrocytic expression of these transporters has been described, which could also present another barrier and contribute to reduced ASD uptake in epileptic tissue (75, 88).

##### Transport of ASDs by Efflux Transporters

Conclusive evidence that ASDs are transported by efflux transporters at therapeutic concentrations is considered the weak link in the transporter hypothesis (101). Early studies suggested that several ASDs may be substrates for P-gp and/or MRPs. However, researchers from different studies used different models, methodologies, and analytical methods with different sensitivities which yielded inconsistent results. Researchers who attempted to identify ASDs as substrates of P-gp, MRPs, and/or BCRP mainly used three approaches: transporter-overexpressing cell lines, transporter inhibition in cell lines and/or in animals, and transporter gene knockout mice (82). Each of these approaches has its own strengths and weaknesses. For example, transporter-overexpressing cell lines only allow *in vitro* analysis. Transporter inhibitors may lack specificity and interact with more than one transporter, and knockout mice may show potential compensatory upregulation of other transporters, which may complicate the situation (78). Therefore, all three approaches may need to be used together in one thorough study to obtain conclusive data (78). In addition, compared to chemotherapeutic drugs that are usually high-affinity substrates for P-gp and MRPs, ASDs are weak substrates for the efflux transporters and more easily cross the blood–brain barrier under physiological conditions (12).

###### ASD Transport by P-gp

P-glycoprotein transports a wide range of structurally and functionally diverse compounds, which are primarily hydrophobic and amphipathic compounds (81). Most ASDs are planar lipophilic molecules, and therefore, theoretically many ASDs should be P-gp substrates (11, 80).

The first report of P-gp-mediated transport of an ASD came from Tishler et al. (77), who reported lower steady-state intracellular phenytoin concentrations in MDR1-expressing neuroectodermal cells as compared to MDR1-negative cells. P-gp-mediated phenytoin transport was also demonstrated *in vivo* using brain microdialysis in normal rats after administration of P-gp inhibitors (79), in rats with SE-induced P-gp upregulation (44), and in *mdr1a/b* knockout mice (102). Phenobarbital, lamotrigine, felbamate, and oxcarbazepine were shown to be transported by P-gp in rat brain microdialysis studies using verapamil as a P-gp inhibitor (103, 104). In contrast, one study using *mdr1a* knockout mice and wild-type control mice showed that out of the seven commonly used ASDs (phenobarbital, phenytoin, carbamazepine, vigabatrin, lamotrigine, gabapentin, and topiramate), only topiramate appeared to be a P-gp substrate (82). However, remaining *mdr1b* expression and potential compensatory upregulation of other efflux transporters in *mdr1a* knockout mice could be limitations of the study (82).

Previous studies on P-gp-mediated transport of carbamazepine yielded inconsistent results (63). Owen et al. (105) concluded that carbamazepine was not a substrate for P-gp based on results from experiments with *mdr1a/b* knockout mice, P-gp-overexpressing Caco-2 cells, and flow cytometry in human lymphocytes using rhodamine 123. In contrast, two other studies, one using *mdr1a/b* knockout mice and the other using *in vivo* microdialysis with verapamil, supported that P-gp transports carbamazepine (102, 106). Data from another microdialysis study in rat suggest that P-gp does not transport levetiracetam (107). Baltes et al. (108) demonstrated that P-gp does also not transport valproic acid by using efflux assays with transfected MDCKII (dog kidney) cells and LLC-PK1 (pig kidney) cells and rat brain microdialysis with the P-gp inhibitors verapamil and tariquidar.

While most of the earlier studies focused on rodent transporters, later and more recent studies used cell lines transfected with human MDR1 or MRPs in order to identify potential species differences in substrate spectrum or transport efficiency of the transporters. Baltes et al. (109) conducted bidirectional transport assays in monolayers of MDCKII and LLC-PK1 cells transfected with complementary DNA containing either MDR1, MRP2, *mdr1a*, or *mdr1b* sequences to study the transport of phenytoin, levetiracetam, and carbamazepine by human and mouse P-gp. The authors concluded that in transfected LLC-PK1 cells, both phenytoin and levetiracetam were transported by mouse P-gp only, while carbamazepine was not transported by human or mouse P-gp (109). Luna-Tortós et al. (110) pointed out that conventional bidirectional transport assays may not be suitable to identify ASDs as P-gp substrates due to the highly permeable nature of most ASDs. Using a modified transport assay (concentration equilibrium transport assay; CETA) which allows evaluating active transport separately from passive permeability, Luna-Tortós et al. detected P-gp transport of phenytoin, phenobarbital, lamotrigine, levetiracetam, and topiramate, but not carbamazepine in MDR1-transfected LLC-PK1 cells (110, 111). Zhang et al. (101) used both the cell monolayer bidirectional assay and CETA in MDR1-transfected MDCKII and LLC-PK1 cells to test if phenytoin, phenobarbital, or ethosuximide were transported by P-gp. Results from the CETA experiments suggested concentration-dependent P-gp transport of phenytoin in both MDCKII-MDR1 and LLC-PK1-MDR1 cells and transport of phenobarbital only in MDCKII-MDR1 cells. In conventional bidirectional transport experiments, however, P-gp-mediated phenytoin transport was minimal, indicating that either cell monolayer permeability may have been too high to detect any differences and/or that CETA has superior sensitivity in studying the active transport of highly permeable compounds (101).

Nonetheless, the results from *in vitro* experiments using cell lines transfected with human proteins should be confirmed using *in vivo* approaches such as PET (112). Verbeek et al. (113) conducted a PET study in rats and concluded that [^11^C]phenytoin was a weak P-gp substrate, as demonstrated by the increase in the brain-to-plasma concentration ratio after P-gp inhibition with tariquidar. In contrast, [^11^C]methylphenobarbital was not shown to be transported by P-gp in a similarly designed PET study in rats and mice (114). At present, data from studies using resected human brain or from clinical trials aimed at identifying if P-gp transports ASDs are limited (96). The only clinical evidence linking overexpression of blood–brain barrier P-gp to reduced ASD brain levels came from a pilot study by Marchi et al. (115). These authors demonstrated an inverse correlation between the brain–plasma concentration ratio of the major active metabolite of oxcarbazepine, 10,11-dihydro-10-hydroxy-5*H*-dibenzo(b,f)azepine-5-carboxamide (10,11-dihydro-10-hydroxycarbamazepine), and the *MDR1* mRNA brain expression levels in resected epileptic tissue from patients with refractory epilepsy (115).

Since different models yield different results, both *in vivo* and *in vitro* data seem to be needed to identify which ASDs are substrates for which transporter. In this regard, by combining the available evidence (as of 2012), Zhang et al. (96) suggested that lamotrigine, oxcarbazepine, phenobarbital, and phenytoin are considered definite P-gp substrates, because P-gp-mediated transport of these ASDs has been supported by both *in vivo* and *in vitro* evidence.

###### ASD Transport by MRPs

Multidrug resistance-associated proteins transport neutral organic drugs and amphiphilic organic anions including drugs conjugated to glutathione, sulfate, glucuronate, and phosphate (85, 86). Thus, it is possible that MRPs transport a number of ASDs and/or their metabolites and limit their access to the brain (32).

Phenytoin transport by MRP1 and/or MRP2 was shown *in vivo* in normal rats using brain microdialysis with the MRP1/2 inhibitor probenecid (116), in TR^−^ mutant rats that lack MRP2 (117), and in rats with seizure-induced MRP1 upregulation (118). Carbamazepine and oxcarbazepine were shown to be substrates of MRP1 and/or MRP2 in microdialysis *in vivo* studies with probenecid (104, 106). Valproic acid was the first ASD found to be a substrate for MRPs in brain endothelial cells (119), but Baltes et al. (108) could not confirm this finding using efflux assays with transfected LLC-PK1 and MDCKII cells and rat brain microdialysis with the MRP inhibitors probenecid and MK571. Similarly, using brain microdialysis in rats, Potschka et al. (107) showed that levetiracetam was not transported by MRP1/2.

Baltes et al. (109) conducted bidirectional transport assays in monolayers of MRP2-transfected MDCKII kidney cells, and none of the ASDs tested (phenytoin, levetiracetam, carbamazepine) was found to be transported by MRP2. Using CETA in MDCKII kidney cells transfected with human MRP1, MRP2, or MRP5, Luna-Tortós et al. reported that none of the ASDs tested (topiramate, valproate, carbamazepine, phenytoin, levetiracetam, lamotrigine, and phenobarbital) was transported by any of those MRPs (111, 112). *In vivo* studies may be needed to confirm the findings from *in vitro* experiments, but few clinical studies have focused on studying the relationship between ASDs and MRPs.

###### ASD Transport by BCRP

Substrate specificity of BCRP significantly overlaps with that of P-gp (120). However, the role of BCRP in ASD resistance is less well studied in comparison to P-gp or the MRPs (121). Using BCRP-transfected MDCKII cells, Cerveny et al. (122) reported that none of the tested ASDs (phenobarbital, phenytoin, ethosuximide, primidone, valproate, carbamazepine, clonazepam, and lamotrigine) was transported by BCRP. However, Nakanishi et al. (123) reported that the brain-to-plasma concentration ratio values of phenobarbital, clobazam, zonisamide, gabapentin, tiagabine, and levetiracetam were higher in *mdr1a/b/Bcrp* triple knockout mice than those in *mdr1a/b* double knockout mice, suggesting the involvement of BCRP in the transport of these ASDs. Subsequently, Römermann et al. (121) reported BCRP transport of lamotrigine using CETA in MDCKII cells transfected with murine *Bcrp* or human *BCRP*, but did not observe transport of phenytoin, phenobarbital, carbamazepine, levetiracetam, topiramate, or valproate. Together, current evidence suggests that most ASDs are not transported by BCRP, though discrepancies exist between *in vitro* and *in vivo* findings (121).

In summary, available data support the transporter substrate status of some ASDs, but overall the evidence is inconsistent and incomprehensive. There is a continued need to systematically investigate the transporter substrate status of ASDs using *in vivo* and *in vitro* models and eventually to confirm the findings in epilepsy patients (96).

##### Mechanisms of Efflux Transporter Upregulation in Epilepsy

An important question that stems from the transporter hypothesis is whether overexpression of efflux transporters at the blood–brain barrier observed in epilepsy is acquired or constitutive. Current evidence suggests that seizures, genetic factors, or a combination of both are likely to be the major contributors to efflux transporter overexpression at the blood–brain barrier in epilepsy (59).

Experimental data mostly from animal studies support that P-gp upregulation in epileptic regions of the brain occurs mainly as a result of seizure activity (124). Rizzi et al. (102) reported *mdr1* mRNA upregulation in brain of mice acutely after kainic acid-induced seizures and in rats with self-sustained seizures after electrically induced SE. Using a rat TLE model in which seizures developed spontaneously after electrically induced SE, van Vliet et al. (84) demonstrated that *mdr1a* mRNA, *mdr1b* mRNA, and P-gp protein levels increased within 1 week after SE. Specifically, chronic epileptic rats had persistent overexpression of *mdr1b* mRNA and P-gp protein in endothelial and glial-like cells of the ventral temporal lobe, with higher P-gp levels in rats that had more seizure activity (84). Levels of *mdr1a* mRNA and P-gp protein levels also increased in whole tissue samples of the temporal hippocampus and the parahippocampal cortex that are involved in epileptogenesis (44). In another study, Bankstahl and Löscher showed overexpression of P-gp protein in brain capillary endothelial cells 48 h after SE in two rat models, the lithium/pilocarpine model and the basolateral amygdala electrical stimulation model (125). van Vliet et al. (126) also reported increased MRP1, MRP2, and BCRP protein expression levels in rat astrocytes and cerebral blood vessels after acute SE and in chronic epilepsy. Similar to the finding with P-gp, overexpression of these transporters was greater in chronic epileptic rats that demonstrated progression of epilepsy (126). Recent research in the field has postulated two main mechanisms leading to efflux transporter overexpression in the brain in epilepsy: (1) ASD-mediated induction of efflux transporters *via* nuclear receptors and (2) seizure-induced signaling causing efflux transporter overexpression.

With regard to the first mechanism, studies on whether ASDs induce efflux transporter overexpression have yielded inconsistent results. Rizzi et al. (102) reported that twice daily intraperitoneal administration of 30 mg/kg phenytoin or 15 mg/kg carbamazepine for 7 days did not alter *mdr1* mRNA expression levels in the mouse hippocampus. However, P-gp expression levels are highest in brain capillaries, and thus, such increases would be masked by using total brain samples due to dilution (brain capillaries make up only 1% of brain volume) (127). Seegers et al. (128) found that giving rats 30 mg/kg phenobarbital or 50 mg/kg phenytoin (following 75 mg/kg on the first day) intraperitoneally daily for 11 days did not significantly increase endothelial or parenchymal P-gp protein expression levels in various brain regions (frontal and parietal cortex, basolateral amygdala, hippocampus, dentate gyrus, piriform cortex, substantia nigra pars reticulata, and cerebellum).

In contrast, in the *Coriaria* lactone-induced rat SE model, Wang-Tilz et al. (129) reported that giving orally 125 mg/kg carbamazepine or 187.5 mg/kg valproic acid daily increased P-gp expression in astrocytes and endothelial cells, particularly in the hippocampus, the temporal, frontal, and parietal lobes of the brain, whereas giving daily 100 mg/kg topiramate or 125 mg/kg lamotrigine orally for 30 days did not affect P-gp expression levels. However, studies have shown that seizures induce brain capillary P-gp expression levels (130, 131). If P-gp levels were already maximally induced in the study of Wang-Tilz et al. (129), one would not expect to see additional increases in P-gp expression levels by ASDs. Consistent with this, Wen et al. (132) reported that 21-day exposure of naïve rats to phenobarbital, carbamazepine, or phenytoin given orally twice daily significantly increased P-gp activity and protein expression levels in capillary endothelial cells in cerebral cortex and hippocampus. The underlying mechanism of this induction was not investigated, but the authors speculated that the observed effect was due to ASD activation of the ligand-activated transcription factors pregnane X receptor and/or constitutive androstane receptor (132). In contrast, Ambroziak et al. (133) did not observe any changes on P-gp expression or activity levels in the GPNT rat brain endothelial cell line and the MDCKII cell line that were exposed to phenobarbital, phenytoin, or carbamazepine. In this regard, it is important to note that ASD-mediated upregulation of drug efflux transporters at the blood–brain barrier and in other tissues does not explain why some patients are resistant to the very first ASD they are given. While this speaks against the theory that ASDs are the main cause for drug resistance due to transporter upregulation, it is possible that ASDs are one contributor, among others, to refractory epilepsy. Clearly, further studies are needed to draw firm conclusions on the effect of ASDs on P-gp expression and activity levels in the brain and their contribution to overall drug resistance in epilepsy.

The second mechanism that has been shown to result in increased efflux transporter expression levels is through recurring seizures. In this regard, Lazarowski et al. (134) showed that daily administration of 3-mercaptopropionic acid (MP) causes daily seizures, which result in a progressive increase of P-gp protein expression at the blood–brain barrier. Furthermore, these researchers showed that the pharmacokinetics of phenytoin are altered in the hippocampus of MP-induced epileptic rats and that treatment with the P-gp inhibitor nimodipine restored normal hippocampal pharmacokinetics of phenytoin resulting in seizure control (135). More recently, the MP-induced seizure model in mice has been presented as a new drug-resistant model that allows screening of drugs at early stages of preclinical trials. After 23 consecutive MP administrations, 100% of animals became resistant to phenytoin and 80% of animals developed resistance to phenobarbital. Resistance was strongly associated with overexpression of P-gp in the cerebral cortex, hippocampus, and striatum. Importantly, resistance to drugs that are not P-gp substrates such as carbamazepine, diazepam, or levetiracetam was not observed (136). Therefore, this new model could be useful for screening novel ASDs that are P-gp substrates and have the potential to control seizures in pharmacoresistant epilepsy.

The molecular signaling mechanism underlying increased efflux transporter expression levels in epilepsy has been studied by our group and others. In this regard, we recently showed that seizure-induced glutamate release triggers a signaling pathway that involves the N-methyl-d-aspartate receptor, cyclooxygenase-2, and the prostanoid E1 receptor, resulting in increased P-gp protein and activity levels at the blood–brain barrier (131, 137–139). In addition, evidence from *in vitro* and *in vivo* rodent studies suggests that targeting this pathway could control P-gp expression and activity levels, and thus, help increase ASD brain penetration and improve ASD efficacy to control seizures in drug-resistant epilepsy (131, 137–139). One study of Salvamoser et al. (140) showed that exposing isolated porcine brain capillaries and human brain capillaries from ASD-resistant patients with FCD to glutamate resulted in reduced BCRP protein expression levels. This finding is in contrast with data from human studies comparing BCRP expression between control and epileptic human brain tissue (89, 90, 92, 95), and unpublished data from our lab clearly demonstrate seizure-induced upregulation of BCRP protein expression and activity levels in brain capillaries from chronic epileptic rats. Considering that Salvamoser et al. neither provided data from dose response nor conducted time course experiments, the observed effect on BCRP in porcine brain capillaries could also be due to glutamate-mediated excitotoxicity. This could also explain the authors’ observation in brain capillaries from epileptic human brain tissue. In this case, capillaries were isolated from resected epileptic brain tissue that has already been exposed to glutamate released during seizures, and thus, adding additional glutamate *ex vivo* will most likely have caused excitotoxicity. Thus, the authors could have misinterpreted glutamate-mediated excitotoxicity as BCRP downregulation.

Together, *in vivo* and *in vitro* experimental data support P-gp upregulation in the epileptic brain as a result of glutamate release and the downstream signaling pathway. Nevertheless, signaling mechanisms that control P-gp and other efflux transporters at the blood–brain barrier have to be first confirmed at the human blood–brain barrier prior to translational development of this strategy (124).

##### Polymorphisms of Efflux Transporters and ASD Response

Hoffmeyer et al. (141) were the first to identify a synonymous C3435T SNP in exon 26 of the human *ABCB1 (MDR1)* gene. In this particular study, individuals with the TT genotype had statistically significantly lower intestinal P-gp protein expression and activity levels as demonstrated by enhanced intestinal uptake of the P-gp substrate digoxin (141). Several other *ABCB1* polymorphisms have been identified later, including a non-synonymous G2677T/A SNP in exon 21 and a synonymous C1236T SNP on exon 12, both of which are thought to be in linkage disequilibrium with C3435T (142) and account for the majority of the *ABCB1* haplotypes along with the C3435T SNP (78). Since the first description of the association between the C3435T SNP and P-gp expression and activity levels, numerous studies have been conducted in an attempt to replicate the results or identify other relevant polymorphisms (143). However, follow-up studies provided conflicting results. For example, Siegmund et al. (144) reported that in healthy Caucasian individuals, none of the genotypes studied, including C3435T, G2677T/A, and other putatively functional SNPs, significantly affected duodenal P-gp protein expression levels or P-gp *in vivo* activity.

Similarly, researchers investigating the association between *ABCB1* polymorphisms and response to ASD treatment found inconsistent results. Siddiqui et al. (11) were the first to investigate ASD resistance in relationship to *ABCB1* polymorphisms. In a study with 315 epilepsy patients, the authors reported that patients with refractory epilepsy had a higher frequency of the CC genotype at the C3435T SNP than the TT genotype. However, Tan et al. (145) could not confirm the association between the C3435T SNP and ASD response in epilepsy. Sills et al. (146) studied the association between the C3435T SNP and pharmacoresistance in 400 epilepsy patients and found no significant differences in allele or genotype frequency between ASD responders and non-responders. Tate et al. (60) reported a lack of association between the C3435T SNP with phenytoin or carbamazepine dosing. Similarly, a study investigating the association between the C3435T polymorphism and drug resistance in 171 Korean patients with epilepsy yielded a negative result (147). Shahwan et al. (148) studied 440 Irish patients with epilepsy and they also could not detect significant associations between ASD resistance and C3435T or seven other functional variants in the *ABCB1* gene.

Using a gene-wide approach, Kwan et al. (142) genotyped 12 tagging and candidate SNPs of *ABCB1* in 464 Chinese patients with epilepsy and revealed significant associations between drug resistance and the intronic polymorphism rs3789243, the coding polymorphism G2677T/A, and haplotypes containing two polymorphisms. In contrast, Leschziner et al. (149) found no significant association between multidrug resistance and C3435T, G2677T/A, C1236T, or a set of tagging SNPs that describe common variations in *ABCB1* in a case–control study with 149 Caucasian epilepsy patients.

Such discrepancies in study results could imply that there is no true association between the *ABCB1* C3435T polymorphism with ASD resistance in epilepsy. An alternative explanation could be that the association was masked by confounding factors such as heterogeneity in the types of ASDs used in the studies, because not all ASDs are P-gp substrates or transported to the same extent (8, 148). Differences in seizure types and definitions of ASD resistance also add to the overall complexity (143, 148). Nevertheless, results from some recent meta-analyses demonstrate that negative findings persist even after controlling for some of the confounding factors. In this regard, Bournissen et al. (150) conducted a meta-analysis of 11 case–control studies (total of 3,371 patients) and investigated the relationship between *ABCB1* C3435T polymorphisms and ASD response. The authors did not find a significant association between the *ABCB1* C3435T SNP and ASD response (odds ratio 1.15; 95% confidence interval 0.78–1.70; *p* = 0.48). Stratification of studies by the ethnicity of the subjects yielded similar results. A meta-analysis conducted by Haerian et al. (151) included 22 genetic association studies (total of 6,755 patients) and also did not identify a significant association between *ABCB1* C3435T polymorphisms and ASD response (odds ratio 1.06, 95% confidence interval 0.98–1.14, *p* = 0.12). Stratified subgroup meta-analyses based on the new definition of drug-resistant epilepsy proposed by the ILAE and based on ethnicity did not reveal any significant associations either (151). Thus, Haerian et al. (152) conducted another meta-analysis to evaluate the association between the *ABCB1* C1236T, G2677T/A, and C3435T loci and ASD response. A total of 26 publications (*n* = 7,831 patients in total) were included for a haplotype meta-analysis, which did not reveal any significant correlation of the polymorphisms and their haplotypes with ASD response either in the general population or in individual ethnic groups. Nevertheless, the authors pointed out that the available data did not allow subgroup analyses based on other confounders, such as types of ASDs used or types of epilepsy (152). Thus, an association between *ABCB1* polymorphisms and P-gp expression and activity levels in patients with refractory epilepsy needs to be confirmed in brain tissue first before the role of *ABCB1* polymorphisms in ASD resistance can be accepted (153). If there was conclusive evidence for C3435T genotype-dependent P-gp expression at the blood–brain barrier, a lack of association between *ABCB1* polymorphisms and ASD response could potentially negate the role P-gp plays in refractory epilepsy (146). Nevertheless, at present there is inadequate evidence supporting the relationship between *ABCB1* polymorphisms and brain *ABCB1* mRNA or P-gp protein expression levels in refractory epilepsy (92, 142, 153).

Even less studied is the role of how *ABCC2* polymorphisms could affect pharmacoresistance in epilepsy, and studies published so far have yielded inconsistent results. In two recently published meta-analysis studies, the researchers investigated the relationship between three common *ABCC2* SNPs (c.-24C>T, c.1249G>A, and c.3972C>T) and ASD response and found a significant association between ASD resistance and c.-24C>T, but not with the other two SNPs (154, 155). However, authors of both reports noted some limitations to their findings, including ethnicity differences in the identified association and variability in how ASD resistance was defined among the studies (154, 155). In contrast, two other meta-analyses identified a significant association between *ABCC2* c.1249G>A and pharmacoresistance (156, 157). The discrepancy in results could be explained by the heterogeneity in the enrolled studies, and thus, current findings need to be confirmed with larger well-designed studies (155).

##### Overcoming Pharmacoresistance with Transporter Inhibitors

One potential strategy to overcome ASD resistance is by directly inhibiting the efflux transporters assumed to be in part responsible for this phenomenon. For P-gp, there are four generations of inhibitors (158). First-generation inhibitors are non-specific for P-gp, such as cyclosporine A and verapamil (2). Second-generation inhibitors [e.g., PSC833 (valspodar), a cyclosporine A analog] are more specific for P-gp, but they still interfere with cytochrome CYP3A4 metabolizing enzyme (2). Third-generation P-gp inhibitors are P-gp-specific and do not interfere with drug metabolizing enzymes (2, 158). Tariquidar (XR9576) in particular is a non-competitive P-gp inhibitor with greater affinity for P-gp than its substrates (20). Finally, fourth-generation P-gp inhibitors (e.g., the cyclic peptide QZ59SE and the natural compounds lamellarin and gomisin A) display low toxicity but high selectivity and potency are currently under development and evaluated for their use in humans (158, 159). MRP inhibitors include probenecid, MK-571, and LY402913 (2). Probenecid effectively inhibits MRPs, especially MRP1 and MRP2 (116).

Experimental data support the concomitant use of P-gp/MRP inhibitors with ASDs as a strategy to increase anticonvulsant brain uptake and efficacy and overcome pharmacoresistance in animal models. Clinckers et al. (104) demonstrated in an *in vivo* microdialysis study that inhibition of P-gp/MRPs using verapamil/probenecid counteracted pharmacoresistance to oxcarbazepine in rats that had experienced pilocarpine-induced seizures. Brandt et al. (47) conducted a study with TLE rats that were divided into two groups based on their response/non-response to phenobarbital at the maximum tolerated doses and found that tariquidar completely counteracted pharmacoresistance. In a similar study, van Vliet et al. (48) first demonstrated that therapeutic doses of phenytoin only partially controlled seizures in chronic epileptic rats where P-gp levels were upregulated in the ventral hippocampus and entorhinal cortex, which was determined by Western blotting of the homogenized brain areas. When coadministered with tariquidar, phenytoin brain concentrations significantly increased and seizures were almost completely controlled (48).

Verapamil, nifedipine, and diltiazem have also been coadministered with ASDs to inhibit P-gp and been evaluated for their effect in increasing ASD brain levels and consequently reducing seizures in patients in clinical practice. Because calcium channel blockers can have intrinsic anticonvulsant activity and inhibitory effect on CYP3A4, it could be difficult to differentiate the effect on P-gp inhibition (2, 160). Several case reports show that adding verapamil to an ASD regimen improved seizure control (160–162). One pilot non-placebo-controlled open-label study in 19 adult patients with refractory TLE found that adding verapamil (120 mg daily in 13 patients and 240 mg daily in 6 patients) to the existing ASD treatment improved seizure control in a dose-dependent manner; in seven patients seizure frequency was reduced by at least 50% (163). In the first randomized, double-blinded placebo-controlled trial that was conducted to evaluate the safety and efficacy of once daily 240 mg verapamil as an add-on therapy in refractory epilepsy patients with focal onset seizures, no statistically significant decrease in seizure frequency was observed in the 12 patients who finished the study; none of the patients achieved 50% or more seizure reduction (164). In this study, adverse effects unique to the verapamil group included skin rashes and feet edema, while no cardiovascular adverse effects were reported. A more recent non-placebo-controlled open-label study explored the efficacy of low-dose verapamil (20 mg three times daily) as adjunctive treatment in refractory epilepsy (165). The authors reported that 10 out of 19 patients who remained in the study achieved 50% or more seizure reduction, and none of the patients experienced cardiovascular or hemodynamic adverse events (165).

Together, the major limitations of these clinical studies are their small patient group size and the use of relatively unspecific P-gp inhibitors (e.g., verapamil), and thus, no firm conclusion about the efficacy of add-on P-gp inhibitors in refractory epilepsy can be drawn at present. This is especially true given the discrepancy in findings from open-label and double-blinded studies.

##### Summary

Sisodiya (6) proposed that a mechanism causing refractory epilepsy needs to be involved in ASD resistance with appropriate functionality and presence in the epileptogenic brain region, and counteracting such a mechanism should reduce refractoriness. In the rodent model, overexpression of P-gp has been observed in epileptic brain tissue, and such overexpression correlates with reduced brain ASD concentrations. Indeed, ASD-resistant rats have higher brain P-gp protein expression levels than ASD-responsive rats, and P-gp inhibition with a specific inhibitor, such as tariquidar, counteracts ASD resistance (59, 166). However, whether such findings from rodent studies can be extrapolated to refractory epilepsy in human patients is unclear (59, 166). It is also unclear if seizure-induced P-gp upregulation at the blood–brain barrier has clinically relevant effects on ASD brain delivery and ultimately on ASD efficacy in epilepsy patients, or if P-gp upregulation is no more than an epiphenomenon of uncontrolled seizures (45).

*In vitro* evidence shows that most ASDs are weak substrates of human P-gp at best (167), but it has also been argued that significant overexpression of multidrug transporters may still restrict ASD access to epileptic neurons *in vivo* (45). On the other hand, as revealed by several meta-analyses, the transporter hypothesis is not supported by genetic association studies (167). Clinical evidence supporting efflux transporter-mediated ASD transport in the human brain has not been demonstrated yet (166). Recent studies utilizing PET/MR imaging, however, demonstrate for the first time increased P-gp transport activity in patients with drug-resistant epilepsy and that seizure reduction after surgery leads to a decrease in P-gp overactivity (100, 168). Together, these patient data suggest that an optimal outcome after surgery is associated with a reduction in P-gp transport activity and that P-gp overexpression could serve as a surrogate marker for drug-resistant epilepsy.

In order to fully assess if P-gp upregulation has any relevant consequences on pharmacoresistance, studying P-gp expression in brain tissue from both ASD-responsive and ASD-resistant patients and/or conducting PET imaging using P-gp substrates or inhibitors in patients would be critical (45, 166). At present, aspects of the transporter hypothesis are still controversial, and further research is needed to determine the clinical relevance of efflux transporter overexpression at the blood–brain barrier.

## Conclusion

Despite the introduction of newer generations of ASDs, pharmacoresistance remains one of the biggest challenges in epilepsy treatment. In this review article, we summarize various theories that have been proposed to explain the mechanism(s) underlying refractory epilepsy with an emphasis on the transporter hypothesis.

The pharmacokinetic hypothesis is supported by case reports that describe subtherapeutic ASD plasma levels in refractory patients, but additional substantiating evidence from animal or human studies is lacking. The neural network hypothesis was inspired by molecular evidence showing the existence of signaling molecules that guide the abnormal growth of axons in epilepsy, but this hypothesis is limited by its inability to account for the occurrence of pharmacoresistance in some but not all epilepsy patients. The intrinsic severity hypothesis is supported by the clinical finding that high frequency of pretreatment seizures is associated with refractoriness, but it fails to explain the complex temporal patterns of ASD resistance in some patients, and a mechanistic explanation behind this hypothesis is also lacking. The gene variant hypothesis is supported by some identified associations between gene variations and ASD resistance, but study findings are often inconsistent and need to be confirmed in larger populations. The strongest evidence for the target hypothesis exists for the loss of use-dependent sodium channel blockade by carbamazepine, but beyond this observation its general utility is limited. Finally, as the most cited hypothesis of refractory epilepsy, the transporter hypothesis is strongly supported by evidence of efflux transporter overexpression at the blood–brain-barrier, but other aspects of the hypothesis remain controversial, especially the clinical relevance of efflux transporter overexpression and the transporter substrate status of many ASDs.

It is clear from current evidence that pharmacoresistance in epilepsy is a multifactorial phenomenon, but based on existing evidence more work is needed to reinforce and integrate the current theories with the ultimate goal of guiding the development of better epilepsy therapies.

## Future Perspectives

### Current Status and Future Development of Treatment Guidelines

The American Academy of Neurology and the American Epilepsy Society guidelines on the treatment of refractory epilepsy were last updated in 2004. These guidelines conclude that all newer ASDs evaluated (gabapentin, lamotrigine, topiramate, tiagabine, oxcarbazepine, levetiracetam, and zonisamide) are appropriate for adjunctive therapy in refractory partial epilepsy in adults (7). However, such recommendations were made in the absence of head-to-head clinical trials that were rationally designed to evaluate the efficacy of two or more ASDs at comparable doses (7). Two other sets of guidelines by the American Academy of Neurology published in 2003 and 2013, respectively, conclude that anteromesial temporal lobe resection in patients with disabling complex partial seizures is more beneficial than continuing pharmacotherapy, and that vagus nerve stimulation is possibly useful for treating children with epilepsy and patients with Lennox–Gastaut syndrome (169, 170). These treatment guidelines recognize the limitations of current treatment options and the scarcity of quality evidence for treating refractory epilepsy. Nevertheless, in addition to incorporating recent clinical evidence, future treatment guidelines need to place more emphasis on personalizing the therapy of patients with refractory epilepsy. In this regard, factors specific to individual patients such as disease etiology, medical history, drug response, temporal patterns of refractoriness, as well as the multifactorial nature of pharmacoresistance need to be taken into account to improve therapy of patients with refractory epilepsy.

### Further Development of Current Hypotheses

Each of the current hypotheses has its limitations, and although each individual theory is applicable to a subgroup of patients, some of these mechanisms may overlap in patients (59). Specifically, it has been proposed that the target hypothesis and the transporter hypothesis are not mutually exclusive and that one mechanism could be predominant for some ASDs but not for others. For example, Remy and Beck (63) proposed that the target mechanism plays a major role in resistance to carbamazepine, as there is conflicting evidence on its P-gp substrate status.

Although the majority of the literature focuses on the transporter hypothesis, further evidence on the clinical relevance of efflux transporter overexpression in refractory epilepsy is still needed. PET studies using P-gp ligands can be used to investigate how P-gp expression and activity is changed in epilepsy and potentially be used to identify patients who can benefit from the use of P-gp inhibitors in the future (17). Until more data become available, it is fair to say that transporter overexpression is most likely not the only factor that plays in ASD resistance and that the best evidence available only supports the plausibility for the clinical role of efflux transporters in refractory epilepsy.

### Treatment Strategies

Based on the transporter hypothesis, one strategy to counteract pharmacoresistance in epilepsy is the adjunctive use of P-gp inhibitors (59). However, the use of P-gp-specific inhibitors is not without concerns as systemic inhibition of P-gp could increase plasma concentrations of drugs and toxins, potentially leading to systemic toxicity (20, 44). The use of a non-specific P-gp inhibitor, such as verapamil, can be limited by its effect on heart rate and blood pressure (160). Though one small open-label study showed that low-dose verapamil was well tolerated (165), this finding still needs to be confirmed in larger double-blinded studies. Another approach we and others suggested is modulating transporter regulation in epilepsy without affecting basal transporter expression and function (131, 137–139). Other strategies include developing new ASDs that are not substrates of efflux transporters (102) and bypassing these transporters using targeted delivery systems (12). Intranasal administration of ASDs has been proposed, but more pharmacokinetic evidence on whether intranasal administration enhances brain delivery of drugs is needed. Intracerebral administration is another option, but the invasive nature of the method limits its application (124).

One important approach to improve the prognosis of epilepsy is to develop new ASDs with greater efficacy, such as by targeting mechanisms unaffected by current ASDs (14, 171). Consequently, there is a need to enhance the understanding of the neurobiological mechanisms underlying ASD resistance in patients and to identify and test novel treatments using various models, including animal models of refractory epilepsy (14, 17). In addition, efforts should be made to search for drugs able to interfere with the progression of epilepsy or hinder neurodegeneration (17).

Several non-pharmacological strategies are currently under development. Stem cell-based therapies and gene therapy are promising strategies, but they have not been tested in clinical trials for epilepsy (172, 173). Potential mechanisms of gene therapy include inhibiting neuronal hyperexcitability, promoting neuronal survival, and facilitating circuit repair by transduction of endogenous cells and expression of modulators or neurotrophic factors. Stem cell-based therapies can be used to replace damaged or dead neurons, provide trophic support to facilitate neuronal survival and repair, or act as a platform for *ex vivo* gene therapy where transplanted neurons are genetically modified to produce therapeutic substances (172).

Drug resistance is one of the most serious problems in epilepsy treatment, and much effort has been made to elucidate the underlying multifactorial mechanisms. In the near future, as we gain more evidence on the proposed hypotheses, we may anticipate further application of treatment strategies that are developed from current understanding of drug resistance, as well as other pharmacological and non-pharmacological approaches that aim to inhibit epileptogenesis and neurodegeneration.

## Author Contributions

FT drafted the outline, wrote the first draft of the manuscript, and revised the manuscript. AH drafted the figures, wrote parts of the manuscript, and revised the figures and manuscript. BB drafted the outline, wrote parts of the manuscript, and revised the figures and manuscript.

## Conflict of Interest Statement

The authors declare that the research was conducted in the absence of any commercial or financial relationships that could be construed as a potential conflict of interest.

## References

[1] Ngugi AK Kariuki SM Bottomley C Kleinschmidt I Sander JW Newton CR. Incidence of epilepsy: a systematic review and meta-analysis. Neurology (2011) 77:1005–12. 10.1212/WNL.0b013e31822cfc90 21893672 PMC3171955

[2] Loscher W Potschka H Role of multidrug transporters in pharmacoresistance to antiepileptic drugs. J Pharmacol Exp Ther (2002) 301:7–14. 10.1124/jpet.301.1.7 11907151

[3] Mohanraj R Brodie MJ. Early predictors of outcome in newly diagnosed epilepsy. Seizure (2013) 22:333–44. 10.1016/j.seizure.2013.02.002 23583115

[4] Kwan P Brodie MJ. Early identification of refractory epilepsy. N Engl J Med (2000) 342:314–9. 10.1056/NEJM200002033420503 10660394

[5] French JA. Refractory epilepsy: one size does not fit all. Epilepsy Curr (2006) 6:177–80. 10.1111/j.1535-7511.2006.00137.x 17260051 PMC1783491

[6] Sisodiya SM. Mechanisms of antiepileptic drug resistance. Curr Opin Neurol (2003) 16:197–201. 10.1097/00019052-200304000-00013 12644749

[7] French JA Kanner AM Bautista J Abou-Khalil B Browne T Harden CL Efficacy and tolerability of the new antiepileptic drugs II: treatment of refractory epilepsy: report of the Therapeutics and Technology Assessment Subcommittee and Quality Standards Subcommittee of the American Academy of Neurology and the American Epilepsy Society. Neurology (2004) 62:1261–73. 10.1212/01.WNL.0000123695.22623.32 15111660

[8] Soranzo N Goldstein DB Sisodiya SM. The role of common variation in drug transporter genes in refractory epilepsy. Expert Opin Pharmacother (2005) 6:1305–12. 10.1517/14656566.6.8.1305 16013981

[9] Schiller Y Najjar Y. Quantifying the response to antiepileptic drugs: effect of past treatment history. Neurology (2008) 70:54–65. 10.1212/01.wnl.0000286959.22040.6e 18166707

[10] Kwan P Arzimanoglou A Berg AT Brodie MJ Allen Hauser W Mathern G Definition of drug resistant epilepsy: consensus proposal by the ad hoc task force of the ILAE Commission on therapeutic strategies. Epilepsia (2010) 51:1069–77. 10.1111/j.1528-1167.2009.02397.x 19889013

[11] Siddiqui A Kerb R Weale ME Brinkmann U Smith A Goldstein DB Association of multidrug resistance in epilepsy with a polymorphism in the drug-transporter gene ABCB1. N Engl J Med (2003) 348:1442–8. 10.1056/NEJMoa021986 12686700

[12] Loscher W Potschka H. Drug resistance in brain diseases and the role of drug efflux transporters. Nat Rev Neurosci (2005) 6:591–602. 10.1038/nrn1728 16025095

[13] French JA. Refractory epilepsy: clinical overview. Epilepsia (2007) 48(Suppl 1):3–7. 10.1111/j.1528-1167.2007.00992.x 17316406

[14] Stephen LJ Brodie MJ. Pharmacotherapy of epilepsy: newly approved and developmental agents. CNS Drugs (2011) 25:89–107. 10.2165/11584860-000000000-00000 21254787

[15] Schmidt D Sillanpaa M. Evidence-based review on the natural history of the epilepsies. Curr Opin Neurol (2012) 25:159–63. 10.1097/WCO.0b013e3283507e73 22274775

[16] Beyenburg S Stavem K Schmidt D. Placebo-corrected efficacy of modern antiepileptic drugs for refractory epilepsy: systematic review and meta-analysis. Epilepsia (2010) 51:7–26. 10.1111/j.1528-1167.2009.02299.x 19744114

[17] Loscher W Schmidt D. Modern antiepileptic drug development has failed to deliver: ways out of the current dilemma. Epilepsia (2011) 52:657–78. 10.1111/j.1528-1167.2011.03024.x 21426333

[18] Sisodiya S. Etiology and management of refractory epilepsies. Nat Clin Pract Neurol (2007) 3:320–30. 10.1038/ncpneuro0521 17549058

[19] Brodie MJ Barry SJ Bamagous GA Norrie JD Kwan P Patterns of treatment response in newly diagnosed epilepsy. Neurology (2012) 78:1548–54. 10.1212/WNL.0b013e3182563b19 22573629 PMC3348850

[20] Kwan P Brodie MJ. Refractory epilepsy: mechanisms and solutions. Expert Rev Neurother (2006) 6:397–406. 10.1586/14737175.6.3.397 16533143

[21] Perry MS Duchowny M. Surgical versus medical treatment for refractory epilepsy: outcomes beyond seizure control. Epilepsia (2013) 54:2060–70. 10.1111/epi.12427 24304432

[22] Laxer KD Trinka E Hirsch LJ Cendes F Langfitt J Delanty N The consequences of refractory epilepsy and its treatment. Epilepsy Behav (2014) 37:59–70. 10.1016/j.yebeh.2014.05.031 24980390

[23] Felton EA Cervenka MC. Dietary therapy is the best option for refractory nonsurgical epilepsy. Epilepsia (2015) 56:1325–9. 10.1111/epi.13075 26198999

[24] Vaccarezza MM Silva WH. Dietary therapy is not the best option for refractory nonsurgical epilepsy. Epilepsia (2015) 56:1330–4. 10.1111/epi.13074 26198854

[25] Kwan P Brodie MJ. Refractory epilepsy: a progressive, intractable but preventable condition? Seizure (2002) 11:77–84. 10.1053/seiz.2002.0593 11945093

[26] Callaghan B Schlesinger M Rodemer W Pollard J Hesdorffer D Allen Hauser W Remission and relapse in a drug-resistant epilepsy population followed prospectively. Epilepsia (2011) 52:619–26. 10.1111/j.1528-1167.2010.02929.x 21269287 PMC3147304

[27] Neligan A Bell GS Elsayed M Sander JW Shorvon SD. Treatment changes in a cohort of people with apparently drug-resistant epilepsy: an extended follow-up. J Neurol Neurosurg Psychiatry (2012) 83:810–3. 10.1136/jnnp-2011-302085 22733083

[28] Neligan A Bell GS Sander JW Shorvon SD. How refractory is refractory epilepsy? Patterns of relapse and remission in people with refractory epilepsy. Epilepsy Res (2011) 96:225–30. 10.1016/j.eplepsyres.2011.06.004 21724372

[29] Briggs DE French JA. What makes epilepsy drug refractory? Expert Rev Neurother (2003) 3:127–31. 10.1586/14737175.3.1.127 19810855

[30] Rogawski MA Johnson MR. Intrinsic severity as a determinant of antiepileptic drug refractoriness. Epilepsy Curr (2008) 8:127–30. 10.1111/j.1535-7511.2008.00272.x 18852835 PMC2566613

[31] Beleza P. Refractory epilepsy: a clinically oriented review. Eur Neurol (2009) 62:65–71. 10.1159/000222775 19521080

[32] Sisodiya SM Lin WR Harding BN Squier MV Thom M. Drug resistance in epilepsy: expression of drug resistance proteins in common causes of refractory epilepsy. Brain (2002) 125:22–31. 10.1093/brain/awf002 11834590

[33] Depondt C. The potential of pharmacogenetics in the treatment of epilepsy. Eur J Paediatr Neurol (2006) 10:57–65. 10.1016/j.ejpn.2005.11.009 16531088

[34] Lazarowski A Czornyj L Lubienieki F Girardi E Vazquez S D’Giano C. ABC transporters during epilepsy and mechanisms underlying multidrug resistance in refractory epilepsy. Epilepsia (2007) 48(Suppl 5):140–9. 10.1111/j.1528-1167.2007.01302.x 17910594

[35] Lazarowski A Massaro M Schteinschnaider A Intruvini S Sevlever G Rabinowicz A Neuronal MDR-1 gene expression and persistent low levels of anticonvulsants in a child with refractory epilepsy. Ther Drug Monit (2004) 26:44–6. 10.1097/00007691-200402000-00010 14749549

[36] Lazarowski A Sevlever G Taratuto A Massaro M Rabinowicz A. Tuberous sclerosis associated with MDR1 gene expression and drug-resistant epilepsy. Pediatr Neurol (1999) 21:731–4. 10.1016/S0887-8994(99)00074-0 10580886

[37] Vazquez SE D’Giano C Carpintiero S Coronel K Ugarnes G Lazarowski A Increase 99mTc-SESTAMIBI (MIBI) liver clearance could identified epileptic pharmacoresistant patients. A preliminary study. Epilepsia (2004) 45:120.

[38] Iwamoto T Kagawa Y Naito Y Kuzuhara S Okuda M. Clinical evaluation of plasma free phenytoin measurement and factors influencing its protein binding. Biopharm Drug Dispos (2006) 27:77–84. 10.1002/bdd.486 16308884

[39] Paul F Veauthier C Fritz G Lehmann TN Aktas O Zipp F Perioperative fluctuations of lamotrigine serum levels in patients undergoing epilepsy surgery. Seizure (2007) 16:479–84. 10.1016/j.seizure.2007.03.006 17433726

[40] Dalaklioglu S. Evaluating appropriateness of digoxin, carbamazepine, valproic acid, and phenytoin usage by therapeutic drug monitoring. Clin Lab (2013) 59:325–31. 10.7754/Clin.Lab.2012.120425 23724621

[41] Fagiolino P Vazquez M Maldonado C Ruiz ME Volonte MG Orozco-Suarez S Usefulness of salivary drug monitoring for detecting efflux transporter overexpression. Curr Pharm Des (2013) 19:6701–8. 10.2174/13816128113199990368 23530513

[42] Kerb R Aynacioglu AS Brockmoller J Schlagenhaufer R Bauer S Szekeres T The predictive value of MDR1, CYP2C9, and CYP2C19 polymorphisms for phenytoin plasma levels. Pharmacogenomics J (2001) 1:204–10. 10.1038/sj.tpj.6500025 11908757

[43] Simon C Stieger B Kullak-Ublick GA Fried M Mueller S Fritschy JM Intestinal expression of cytochrome P450 enzymes and ABC transporters and carbamazepine and phenytoin disposition. Acta Neurol Scand (2007) 115:232–42. 10.1111/j.1600-0404.2006.00761.x 17376120

[44] van Vliet EA van Schaik R Edelbroek PM Voskuyl RA Redeker S Aronica E Region-specific overexpression of P-glycoprotein at the blood-brain barrier affects brain uptake of phenytoin in epileptic rats. J Pharmacol Exp Ther (2007) 322:141–7. 10.1124/jpet.107.121178 17392402

[45] Loscher W Luna-Tortós C Römermann K Fedrowitz M. Do ATP-binding cassette transporters cause pharmacoresistance in epilepsy? Problems and approaches in determining which antiepileptic drugs are affected. Curr Pharm Des (2011) 17:2808–28. 10.2174/138161211797440212 21827408

[46] Volk HA Löscher W. Multidrug resistance in epilepsy: rats with drug-resistant seizures exhibit enhanced brain expression of P-glycoprotein compared with rats with drug-responsive seizures. Brain (2005) 128:1358–68. 10.1093/brain/awh437 15716304

[47] Brandt C Bethmann K Gastens AM Loscher W. The multidrug transporter hypothesis of drug resistance in epilepsy: proof-of-principle in a rat model of temporal lobe epilepsy. Neurobiol Dis (2006) 24:202–11. 10.1016/j.nbd.2006.06.014 16928449

[48] van Vliet EA van Schaik R Edelbroek PM Redeker S Aronica E Wadman WJ Inhibition of the multidrug transporter P-glycoprotein improves seizure control in phenytoin-treated chronic epileptic rats. Epilepsia (2006) 47:672–80. 10.1111/j.1528-1167.2006.00496.x 16650133

[49] Schmidt D Haenel F. Therapeutic plasma levels of phenytoin, phenobarbital, and carbamazepine: individual variation in relation to seizure frequency and type. Neurology (1984) 34:1252–5. 10.1212/WNL.34.9.1252 6540414

[50] Johannessen SI Battino D Berry DJ Bialer M Kramer G Tomson T Therapeutic drug monitoring of the newer antiepileptic drugs. Ther Drug Monit (2003) 25:347–63. 10.1097/00007691-200306000-00016 12766564

[51] Fang M Xi ZQ Wu Y Wang XF. A new hypothesis of drug refractory epilepsy: neural network hypothesis. Med Hypotheses (2011) 76:871–6. 10.1016/j.mehy.2011.02.039 21429675

[52] Rogawski MA. The intrinsic severity hypothesis of pharmacoresistance to antiepileptic drugs. Epilepsia (2013) 54(Suppl 2):33–40. 10.1111/epi.12182 23646969

[53] Berg AT Shinnar S Levy SR Testa FM Smith-Rapaport S Beckerman B Early development of intractable epilepsy in children: a prospective study. Neurology (2001) 56:1445–52. 10.1212/WNL.56.11.1445 11402099

[54] Hitiris N Mohanraj R Norrie J Sills GJ Brodie MJ. Predictors of pharmacoresistant epilepsy. Epilepsy Res (2007) 75:192–6. 10.1016/j.eplepsyres.2007.06.003 17628429

[55] Brodie MJ. Road to refractory epilepsy: the Glasgow story. Epilepsia (2013) 54(Suppl 2):5–8. 10.1111/epi.12175 23646962

[56] Musicco M Beghi E Solari A Viani F Treatment of first tonic-clonic seizure does not improve the prognosis of epilepsy. First Seizure Trial Group (FIRST Group). Neurology (1997) 49:991–8. 10.1212/WNL.49.4.991 9339678

[57] Camfield C Camfield P Gordon K Dooley J Does the number of seizures before treatment influence ease of control or remission of childhood epilepsy? Not if the number is 10 or less. Neurology (1996) 46:41–4. 10.1212/WNL.46.1.41 8559418

[58] Marson A Jacoby A Johnson A Kim L Gamble C Chadwick D Immediate versus deferred antiepileptic drug treatment for early epilepsy and single seizures: a randomised controlled trial. Lancet (2005) 365:2007–13. 10.1016/S0140-6736(05)66694-9 15950714

[59] Schmidt D Loscher W. New developments in antiepileptic drug resistance: an integrative view. Epilepsy Curr (2009) 9:47–52. 10.1111/j.1535-7511.2008.01289.x 19421380 PMC2673406

[60] Tate SK Depondt C Sisodiya SM Cavalleri GL Schorge S Soranzo N Genetic predictors of the maximum doses patients receive during clinical use of the anti-epileptic drugs carbamazepine and phenytoin. Proc Natl Acad Sci U S A (2005) 102:5507–12. 10.1073/pnas.0407346102 15805193 PMC556232

[61] van der Weide J Steijns LS van Weelden MJ de Haan K The effect of genetic polymorphism of cytochrome P450 CYP2C9 on phenytoin dose requirement. Pharmacogenetics (2001) 11:287–91. 10.1097/00008571-200106000-00002 11434505

[62] Ufer M Mosyagin I Muhle H Jacobsen T Haenisch S Hasler R Non-response to antiepileptic pharmacotherapy is associated with the ABCC2 -24C>T polymorphism in young and adult patients with epilepsy. Pharmacogenet Genomics (2009) 19:353–62. 10.1097/FPC.0b013e328329940b 19415824

[63] Remy S Beck H Molecular and cellular mechanisms of pharmacoresistance in epilepsy. Brain (2006) 129:18–35. 10.1093/brain/awh682 16317026

[64] Kwan P Poon WS Ng HK Kang DE Wong V Ng PW Multidrug resistance in epilepsy and polymorphisms in the voltage-gated sodium channel genes SCN1A, SCN2A, and SCN3A: correlation among phenotype, genotype, and mRNA expression. Pharmacogenet Genomics (2008) 18:989–98. 10.1097/FPC.0b013e3283117d67 18784617

[65] Tate SK Singh R Hung CC Tai JJ Depondt C Cavalleri GL A common polymorphism in the SCN1A gene associates with phenytoin serum levels at maintenance dose. Pharmacogenet Genomics (2006) 16:721–6. 10.1097/01.fpc.0000230114.41828.73 17001291

[66] Abe T Seo T Ishitsu T Nakagawa T Hori M Nakagawa K. Association between SCN1A polymorphism and carbamazepine-resistant epilepsy. Br J Clin Pharmacol (2008) 66:304–7. 10.1111/j.1365-2125.2008.03203.x 18489610 PMC2492927

[67] Lakhan R Kumari R Misra UK Kalita J Pradhan S Mittal B. Differential role of sodium channels SCN1A and SCN2A gene polymorphisms with epilepsy and multiple drug resistance in the North Indian population. Br J Clin Pharmacol (2009) 68:214–20. 10.1111/j.1365-2125.2009.03437.x 19694741 PMC2767285

[68] Kumari R Lakhan R Garg RK Kalita J Misra UK Mittal B. Pharmacogenomic association study on the role of drug metabolizing, drug transporters and drug target gene polymorphisms in drug-resistant epilepsy in a north Indian population. Indian J Hum Genet (2011) 17(Suppl 1):S32–40. 10.4103/0971-6866.80357 21747585 PMC3125053

[69] Abo El Fotoh WM Abd El Naby SA Habib MS ALrefai AA Kasemy ZA. The potential implication of SCN1A and CYP3A5 genetic variants on antiepileptic drug resistance among Egyptian epileptic children. Seizure (2016) 41:75–80. 10.1016/j.seizure.2016.07.005 27498208

[70] Grover S Gupta M Kukreti R. Challenges and recommendations for conducting epidemiological studies in the field of epilepsy pharmacogenetics. Indian J Hum Genet (2011) 17(Suppl 1):S4–11. 10.4103/0971-6866.80351 21747586 PMC3125045

[71] Remy S Gabriel S Urban BW Dietrich D Lehmann TN Elger CE A novel mechanism underlying drug resistance in chronic epilepsy. Ann Neurol (2003) 53:469–79. 10.1002/ana.10473 12666114

[72] Hitiris N Brodie MJ. Modern antiepileptic drugs: guidelines and beyond. Curr Opin Neurol (2006) 19:175–80. 10.1097/01.wco.0000218235.67840.82 16538093

[73] Loup F Wieser HG Yonekawa Y Aguzzi A Fritschy JM. Selective alterations in GABAA receptor subtypes in human temporal lobe epilepsy. J Neurosci (2000) 20:5401–19. 10884325 10.1523/JNEUROSCI.20-14-05401.2000 PMC6772330

[74] Pirker S Schwarzer C Czech T Baumgartner C Pockberger H Maier H Increased expression of GABA(A) receptor beta-subunits in the hippocampus of patients with temporal lobe epilepsy. J Neuropathol Exp Neurol (2003) 62:820–34. 10.1093/jnen/62.8.820 14503638

[75] Sisodiya SM Martinian L Scheffer GL van der Valk P Scheper RJ Harding BN Vascular colocalization of P-glycoprotein, multidrug-resistance associated protein 1, breast cancer resistance protein and major vault protein in human epileptogenic pathologies. Neuropathol Appl Neurobiol (2006) 32:51–63. 10.1111/j.1365-2990.2005.00699.x 16409553

[76] Mao Q Unadkat JD Role of the breast cancer resistance protein (BCRP/ABCG2) in drug transport – an update. AAPS J (2015) 17:65–82. 10.1208/s12248-014-9668-6 25236865 PMC4287283

[77] Tishler DM Weinberg KI Hinton DR Barbaro N Annett GM Raffel C. MDR1 gene expression in brain of patients with medically intractable epilepsy. Epilepsia (1995) 36:1–6. 10.1111/j.1528-1157.1995.tb01657.x 8001500

[78] Kwan P Brodie MJ. Potential role of drug transporters in the pathogenesis of medically intractable epilepsy. Epilepsia (2005) 46:224–35. 10.1111/j.0013-9580.2005.31904.x 15679503

[79] Potschka H Loscher W In vivo evidence for P-glycoprotein-mediated transport of phenytoin at the blood-brain barrier of rats. Epilepsia (2001) 42:1231–40. 10.1046/j.1528-1157.2001.01901.x 11737157

[80] Dombrowski SM Desai SY Marroni M Cucullo L Goodrich K Bingaman W Overexpression of multiple drug resistance genes in endothelial cells from patients with refractory epilepsy. Epilepsia (2001) 42:1501–6. 10.1046/j.1528-1157.2001.12301.x 11879359

[81] Chufan EE Sim HM Ambudkar SV. Molecular basis of the polyspecificity of P-glycoprotein (ABCB1): recent biochemical and structural studies. Adv Cancer Res (2015) 125:71–96. 10.1016/bs.acr.2014.10.003 25640267 PMC7709800

[82] Sills GJ Kwan P Butler E de Lange EC van den Berg DJ Brodie MJ. P-glycoprotein-mediated efflux of antiepileptic drugs: preliminary studies in mdr1a knockout mice. Epilepsy Behav (2002) 3:427–32. 10.1016/S1525-5050(02)00511-5 12609264

[83] Ashraf T Kao A Bendayan R. Functional expression of drug transporters in glial cells: potential role on drug delivery to the CNS. Adv Pharmacol (2014) 71:45–111. 10.1016/bs.apha.2014.06.010 25307214

[84] van Vliet E Aronica E Redeker S Marchi N Rizzi M Vezzani A Selective and persistent upregulation of mdr1b mRNA and P-glycoprotein in the parahippocampal cortex of chronic epileptic rats. Epilepsy Res (2004) 60:203–13. 10.1016/j.eplepsyres.2004.06.005 15380564

[85] Zhou SF Wang LL Di YM Xue CC Duan W Li CG Substrates and inhibitors of human multidrug resistance associated proteins and the implications in drug development. Curr Med Chem (2008) 15:1981–2039. 10.2174/092986708785132870 18691054

[86] Keppler D. Multidrug resistance proteins (MRPs, ABCCs): importance for pathophysiology and drug therapy. Handb Exp Pharmacol (2011) 201:299–323. 10.1007/978-3-642-14541-4_8 21103974

[87] Nies AT Jedlitschky G Konig J Herold-Mende C Steiner HH Schmitt HP Expression and immunolocalization of the multidrug resistance proteins, MRP1-MRP6 (ABCC1-ABCC6), in human brain. Neuroscience (2004) 129:349–60. 10.1016/j.neuroscience.2004.07.051 15501592

[88] Aronica E Gorter JA Ramkema M Redeker S Ozbas-Gerceker F van Vliet EA Expression and cellular distribution of multidrug resistance-related proteins in the hippocampus of patients with mesial temporal lobe epilepsy. Epilepsia (2004) 45:441–51. 10.1111/j.0013-9580.2004.57703.x 15101825

[89] Aronica E Gorter JA Redeker S van Vliet EA Ramkema M Scheffer GL Localization of breast cancer resistance protein (BCRP) in microvessel endothelium of human control and epileptic brain. Epilepsia (2005) 46:849–57. 10.1111/j.1528-1167.2005.66604.x 15946326

[90] Liu JY Thom M Catarino CB Martinian L Figarella-Branger D Bartolomei F Neuropathology of the blood-brain barrier and pharmaco-resistance in human epilepsy. Brain (2012) 135:3115–33. 10.1093/brain/aws147 22750659

[91] Aronica E Gorter JA Jansen GH van Veelen CW van Rijen PC Leenstra S Expression and cellular distribution of multidrug transporter proteins in two major causes of medically intractable epilepsy: focal cortical dysplasia and glioneuronal tumors. Neuroscience (2003) 118:417–29. 10.1016/S0306-4522(02)00992-2 12699778

[92] Vogelgesang S Kunert-Keil C Cascorbi I Mosyagin I Schroder E Runge U Expression of multidrug transporters in dysembryoplastic neuroepithelial tumors causing intractable epilepsy. Clin Neuropathol (2004) 23:223–31. 15581025

[93] Ak H Ay B Tanriverdi T Sanus GZ Is M Sar M Expression and cellular distribution of multidrug resistance-related proteins in patients with focal cortical dysplasia. Seizure (2007) 16:493–503. 10.1016/j.seizure.2007.03.011 17482840

[94] Sun Y Luo X Yang K Sun X Li X Zhang C Neural overexpression of multidrug resistance-associated protein 1 and refractory epilepsy: a meta-analysis of nine studies. Int J Neurosci (2016) 126:308–17. 10.3109/00207454.2015.1015724 26000815

[95] Sisodiya SM Martinian L Scheffer GL van der Valk P Cross JH Scheper RJ Major vault protein, a marker of drug resistance, is upregulated in refractory epilepsy. Epilepsia (2003) 44:1388–96. 10.1046/j.1528-1157.2003.21803.x 14636345

[96] Zhang C Kwan P Zuo Z Baum L. The transport of antiepileptic drugs by P-glycoprotein. Adv Drug Deliv Rev (2012) 64:930–42. 10.1016/j.addr.2011.12.003 22197850

[97] Syvanen S Eriksson J. Advances in PET imaging of P-glycoprotein function at the blood-brain barrier. ACS Chem Neurosci (2013) 4:225–37. 10.1021/cn3001729 23421673 PMC3582299

[98] Langer O Bauer M Hammers A Karch R Pataraia E Koepp MJ Pharmacoresistance in epilepsy: a pilot PET study with the P-glycoprotein substrate R-[(11)C]verapamil. Epilepsia (2007) 48:1774–84. 10.1111/j.1528-1167.2007.01116.x 17484754

[99] Feldmann M Asselin MC Liu J Wang S McMahon A Anton-Rodriguez J P-glycoprotein expression and function in patients with temporal lobe epilepsy: a case-control study. Lancet Neurol (2013) 12:777–85. 10.1016/S1474-4422(13)70109-1 23786896

[100] Shin JW Chu K Shin SA Jung KH Lee ST Lee YS Clinical applications of simultaneous PET/MR imaging using (R)-[11C]-verapamil with cyclosporin A: preliminary results on a surrogate marker of drug-resistant epilepsy. AJNR Am J Neuroradiol (2016) 37:600–6. 10.3174/ajnr.A4566 26585254 PMC7960172

[101] Zhang C Kwan P Zuo Z Baum L. In vitro concentration dependent transport of phenytoin and phenobarbital, but not ethosuximide, by human P-glycoprotein. Life Sci (2010) 86:899–905. 10.1016/j.lfs.2010.04.008 20417647

[102] Rizzi M Caccia S Guiso G Richichi C Gorter JA Aronica E Limbic seizures induce P-glycoprotein in rodent brain: functional implications for pharmacoresistance. J Neurosci (2002) 22:5833–9. 12122045 10.1523/JNEUROSCI.22-14-05833.2002 PMC6757954

[103] Potschka H Fedrowitz M Loscher W. P-Glycoprotein-mediated efflux of phenobarbital, lamotrigine, and felbamate at the blood-brain barrier: evidence from microdialysis experiments in rats. Neurosci Lett (2002) 327:173–6. 10.1016/S0304-3940(02)00423-8 12113905

[104] Clinckers R Smolders I Meurs A Ebinger G Michotte Y. Quantitative in vivo microdialysis study on the influence of multidrug transporters on the blood-brain barrier passage of oxcarbazepine: concomitant use of hippocampal monoamines as pharmacodynamic markers for the anticonvulsant activity. J Pharmacol Exp Ther (2005) 314:725–31. 10.1124/jpet.105.085514 15860570

[105] Owen A Pirmohamed M Tettey JN Morgan P Chadwick D Park BK. Carbamazepine is not a substrate for P-glycoprotein. Br J Clin Pharmacol (2001) 51:345–9. 10.1046/j.1365-2125.2001.01359.x 11318771 PMC2014449

[106] Potschka H Fedrowitz M Loscher W. P-glycoprotein and multidrug resistance-associated protein are involved in the regulation of extracellular levels of the major antiepileptic drug carbamazepine in the brain. Neuroreport (2001) 12:3557–60. 10.1097/00001756-200111160-00037 11733711

[107] Potschka H Baltes S Loscher W Inhibition of multidrug transporters by verapamil or probenecid does not alter blood-brain barrier penetration of levetiracetam in rats. Epilepsy Res (2004) 58:85–91. 10.1016/j.eplepsyres.2003.12.007 15120740

[108] Baltes S Fedrowitz M Tortos CL Potschka H Loscher W Valproic acid is not a substrate for P-glycoprotein or multidrug resistance proteins 1 and 2 in a number of in vitro and in vivo transport assays. J Pharmacol Exp Ther (2007) 320:331–43. 10.1124/jpet.106.102491 17043155

[109] Baltes S Gastens AM Fedrowitz M Potschka H Kaever V Loscher W Differences in the transport of the antiepileptic drugs phenytoin, levetiracetam and carbamazepine by human and mouse P-glycoprotein. Neuropharmacology (2007) 52:333–46. 10.1016/j.neuropharm.2006.07.038 17045309

[110] Luna-Tortós C Fedrowitz M Loscher W. Several major antiepileptic drugs are substrates for human P-glycoprotein. Neuropharmacology (2008) 55:1364–75. 10.1016/j.neuropharm.2008.08.032 18824002

[111] Luna-Tortós C Rambeck B Jurgens UH Loscher W. The antiepileptic drug topiramate is a substrate for human P-glycoprotein but not multidrug resistance proteins. Pharm Res (2009) 26:2464–70. 10.1007/s11095-009-9961-8 19730994

[112] Luna-Tortós C Fedrowitz M Loscher W. Evaluation of transport of common antiepileptic drugs by human multidrug resistance-associated proteins (MRP1, 2 and 5) that are overexpressed in pharmacoresistant epilepsy. Neuropharmacology (2010) 58:1019–32. 10.1016/j.neuropharm.2010.01.007 20080116

[113] Verbeek J Eriksson J Syvanen S Labots M de Lange EC Voskuyl RA [11C]phenytoin revisited: synthesis by [11C]CO carbonylation and first evaluation as a P-gp tracer in rats. EJNMMI Res (2012) 2:36. 10.1186/2191-219X-2-36 22747744 PMC3506555

[114] Mairinger S Bankstahl JP Kuntner C Römermann K Bankstahl M Wanek T The antiepileptic drug mephobarbital is not transported by P-glycoprotein or multidrug resistance protein 1 at the blood-brain barrier: a positron emission tomography study. Epilepsy Res (2012) 100:93–103. 10.1016/j.eplepsyres.2012.01.012 22342565 PMC3778256

[115] Marchi N Guiso G Rizzi M Pirker S Novak K Czech T A pilot study on brain-to-plasma partition of 10,11-dyhydro-10-hydroxy-5H-dibenzo(b,f)azepine-5-carboxamide and MDR1 brain expression in epilepsy patients not responding to oxcarbazepine. Epilepsia (2005) 46:1613–9. 10.1111/j.1528-1167.2005.00265.x 16190932

[116] Potschka H Loscher W Multidrug resistance-associated protein is involved in the regulation of extracellular levels of phenytoin in the brain. Neuroreport (2001) 12:2387–9. 10.1097/00001756-200111160-00037 11496115

[117] Potschka H Fedrowitz M Loscher W. Multidrug resistance protein MRP2 contributes to blood-brain barrier function and restricts antiepileptic drug activity. J Pharmacol Exp Ther (2003) 306:124–31. 10.1124/jpet.103.049858 12663688

[118] Chen YH Wang CC Xiao X Wei L Xu G. Multidrug resistance-associated protein 1 decreases the concentrations of antiepileptic drugs in cortical extracellular fluid in amygdale kindling rats. Acta Pharmacol Sin (2013) 34:473–9. 10.1038/aps.2012.183 23474709 PMC4002787

[119] Huai-Yun H Secrest DT Mark KS Carney D Brandquist C Elmquist WF Expression of multidrug resistance-associated protein (MRP) in brain microvessel endothelial cells. Biochem Biophys Res Commun (1998) 243:816–20. 10.1006/bbrc.1997.8132 9500978

[120] Agarwal S Hartz AM Elmquist WF Bauer B. Breast cancer resistance protein and P-glycoprotein in brain cancer: two gatekeepers team up. Curr Pharm Des (2011) 17:2793–802. 10.1007/s11307-010-0313-1 21827403 PMC3269897

[121] Römermann K Helmer R Loscher W. The antiepileptic drug lamotrigine is a substrate of mouse and human breast cancer resistance protein (ABCG2). Neuropharmacology (2015) 93:7–14. 10.1016/j.neuropharm.2015.01.015 25645391

[122] Cerveny L Pavek P Malakova J Staud F Fendrich Z. Lack of interactions between breast cancer resistance protein (bcrp/abcg2) and selected antiepileptic agents. Epilepsia (2006) 47:461–8. 10.1111/j.1528-1167.2006.00453.x 16529607

[123] Nakanishi H Yonezawa A Matsubara K Yano I. Impact of P-glycoprotein and breast cancer resistance protein on the brain distribution of antiepileptic drugs in knockout mouse models. Eur J Pharmacol (2013) 710:20–8. 10.1016/j.ejphar.2013.03.049 23588114

[124] Potschka H. Modulating P-glycoprotein regulation: future perspectives for pharmacoresistant epilepsies? Epilepsia (2010) 51:1333–47. 10.1111/j.1528-1167.2010.02585.x 20477844

[125] Bankstahl JP Loscher W. Resistance to antiepileptic drugs and expression of P-glycoprotein in two rat models of status epilepticus. Epilepsy Res (2008) 82:70–85. 10.1016/j.eplepsyres.2008.07.007 18760905

[126] van Vliet EA Redeker S Aronica E Edelbroek PM Gorter JA. Expression of multidrug transporters MRP1, MRP2, and BCRP shortly after status epilepticus, during the latent period, and in chronic epileptic rats. Epilepsia (2005) 46:1569–80. 10.1111/j.1528-1167.2005.00250.x 16190927

[127] Pardridge WM. Blood-brain barrier genomics and the use of endogenous transporters to cause drug penetration into the brain. Curr Opin Drug Discov Devel (2003) 6:683–91. 14579518

[128] Seegers U Potschka H Loscher W. Lack of effects of prolonged treatment with phenobarbital or phenytoin on the expression of P-glycoprotein in various rat brain regions. Eur J Pharmacol (2002) 451:149–55. 10.1016/S0014-2999(02)02235-5 12231384

[129] Wang-Tilz Y Tilz C Wang B Tilz GP Stefan H. Influence of lamotrigine and topiramate on MDR1 expression in difficult-to-treat temporal lobe epilepsy. Epilepsia (2006) 47:233–9. 10.1111/j.1528-1167.2006.00414.x 16499746

[130] Potschka H Volk HA Loscher W Pharmacoresistance and expression of multidrug transporter P-glycoprotein in kindled rats. Neuroreport (2004) 15:1657–61. 10.1097/01.wnr.0000134840.10390.a4 15232302

[131] Bauer B Hartz AM Pekcec A Toellner K Miller DS Potschka H. Seizure-induced up-regulation of P-glycoprotein at the blood-brain barrier through glutamate and cyclooxygenase-2 signaling. Mol Pharmacol (2008) 73:1444–53. 10.1124/mol.107.041210 18094072

[132] Wen T Liu YC Yang HW Liu HY Liu XD Wang GJ Effect of 21-day exposure of phenobarbital, carbamazepine and phenytoin on P-glycoprotein expression and activity in the rat brain. J Neurol Sci (2008) 270:99–106. 10.1016/j.jns.2008.02.016 18440557

[133] Ambroziak K Kuteykin-Teplyakov K Luna-Tortós C Al-Falah M Fedrowitz M Loscher W Exposure to antiepileptic drugs does not alter the functionality of P-glycoprotein in brain capillary endothelial and kidney cell lines. Eur J Pharmacol (2010) 628:57–66. 10.1016/j.ejphar.2009.11.051 19958760

[134] Lazarowski A Ramos AJ Garcia-Rivello H Brusco A Girardi E Neuronal and glial expression of the multidrug resistance gene product in an experimental epilepsy model. Cell Mol Neurobiol (2004) 24:77–85. 10.1023/B:CEMN.0000012726.43842.d2 15049512 PMC11529958

[135] Hocht C Lazarowski A Gonzalez NN Auzmendi J Opezzo JA Bramuglia GF Nimodipine restores the altered hippocampal phenytoin pharmacokinetics in a refractory epileptic model. Neurosci Lett (2007) 413:168–72. 10.1016/j.neulet.2006.11.075 17240061

[136] Enrique A Goicoechea S Castano R Taborda F Rocha L Orozco S New model of pharmacoresistant seizures induced by 3-mercaptopropionic acid in mice. Epilepsy Res (2017) 129:8–16. 10.1016/j.eplepsyres.2016.10.012 27875747

[137] Hartz AM Notenboom S Bauer B. Signaling to P-glycoprotein-A new therapeutic target to treat drug-resistant epilepsy? Drug News Perspect (2009) 22:393–7. 10.1358/dnp.2009.22.7.1401354 19890496

[138] Pekcec A Unkruer B Schlichtiger J Soerensen J Hartz AM Bauer B Targeting prostaglandin E2 EP1 receptors prevents seizure-associated P-glycoprotein up-regulation. J Pharmacol Exp Ther (2009) 330:939–47. 10.1124/jpet.109.152520 19494186

[139] Zibell G Unkruer B Pekcec A Hartz AM Bauer B Miller DS Prevention of seizure-induced up-regulation of endothelial P-glycoprotein by COX-2 inhibition. Neuropharmacology (2009) 56:849–55. 10.1016/j.neuropharm.2009.01.009 19371577

[140] Salvamoser JD Avemary J Luna-Munguia H Pascher B Getzinger T Pieper T Glutamate-mediated down-regulation of the multidrug-resistance protein BCRP/ABCG2 in porcine and human brain capillaries. Mol Pharm (2015) 12:2049–60. 10.1021/mp500841w 25898179

[141] Hoffmeyer S Burk O von Richter O Arnold HP Brockmoller J Johne A Functional polymorphisms of the human multidrug-resistance gene: multiple sequence variations and correlation of one allele with P-glycoprotein expression and activity in vivo. Proc Natl Acad Sci U S A (2000) 97:3473–8. 10.1073/pnas.97.7.3473 10716719 PMC16264

[142] Kwan P Wong V Ng PW Lui CH Sin NC Poon WS Gene-wide tagging study of association between ABCB1 polymorphisms and multidrug resistance in epilepsy in Han Chinese. Pharmacogenomics (2009) 10:723–32. 10.2217/pgs.09.32 19450124

[143] Leschziner GD Andrew T Pirmohamed M Johnson MR ABCB1 genotype and PGP expression, function and therapeutic drug response: a critical review and recommendations for future research. Pharmacogenomics J (2007) 7:154–79. 10.1038/sj.tpj.6500413 16969364

[144] Siegmund W Ludwig K Giessmann T Dazert P Schroeder E Sperker B The effects of the human MDR1 genotype on the expression of duodenal P-glycoprotein and disposition of the probe drug talinolol. Clin Pharmacol Ther (2002) 72:572–83. 10.1067/mcp.2002.127739 12426521

[145] Tan NC Heron SE Scheffer IE Pelekanos JT McMahon JM Vears DF Failure to confirm association of a polymorphism in ABCB1 with multidrug-resistant epilepsy. Neurology (2004) 63:1090–2. 10.1212/01.WNL.0000137051.33486.C7 15452306

[146] Sills GJ Mohanraj R Butler E McCrindle S Collier L Wilson EA Lack of association between the C3435T polymorphism in the human multidrug resistance (MDR1) gene and response to antiepileptic drug treatment. Epilepsia (2005) 46:643–7. 10.1111/j.1528-1167.2005.46304.x 15857428

[147] Kim DW Kim M Lee SK Kang R Lee SY. Lack of association between C3435T nucleotide MDR1 genetic polymorphism and multidrug-resistant epilepsy. Seizure (2006) 15:344–7. 10.1016/j.seizure.2006.02.015 16542858

[148] Shahwan A Murphy K Doherty C Cavalleri GL Muckian C Dicker P The controversial association of ABCB1 polymorphisms in refractory epilepsy: an analysis of multiple SNPs in an Irish population. Epilepsy Res (2007) 73:192–8. 10.1016/j.eplepsyres.2006.10.004 17125969

[149] Leschziner GD Andrew T Leach JP Chadwick D Coffey AJ Balding DJ Common ABCB1 polymorphisms are not associated with multidrug resistance in epilepsy using a gene-wide tagging approach. Pharmacogenet Genomics (2007) 17:217–20. 10.1097/01.fpc.0000230408.23146.b1 17460550

[150] Bournissen FG Moretti ME Juurlink DN Koren G Walker M Finkelstein Y. Polymorphism of the MDR1/ABCB1 C3435T drug-transporter and resistance to anticonvulsant drugs: a meta-analysis. Epilepsia (2009) 50:898–903. 10.1111/j.1528-1167.2008.01858.x 19178561

[151] Haerian BS Roslan H Raymond AA Tan CT Lim KS Zulkifli SZ ABCB1 C3435T polymorphism and the risk of resistance to antiepileptic drugs in epilepsy: a systematic review and meta-analysis. Seizure (2010) 19:339–46. 10.1016/j.seizure.2010.05.004 20605481

[152] Haerian BS Lim KS Tan CT Raymond AA Mohamed Z. Association of ABCB1 gene polymorphisms and their haplotypes with response to antiepileptic drugs: a systematic review and meta-analysis. Pharmacogenomics (2011) 12:713–25. 10.2217/pgs.10.212 21391884

[153] Mosyagin I Runge U Schroeder HW Dazert E Vogelgesang S Siegmund W Association of ABCB1 genetic variants 3435C>T and 2677G>T to ABCB1 mRNA and protein expression in brain tissue from refractory epilepsy patients. Epilepsia (2008) 49:1555–61. 10.1111/j.1528-1167.2008.01661.x 18494787

[154] Grover S Kukreti R. A systematic review and meta-analysis of the role of ABCC2 variants on drug response in patients with epilepsy. Epilepsia (2013) 54:936–45. 10.1111/epi.12132 23506516

[155] Qian L Fang S Yan YL Zeng SS Xu ZJ Gong ZC The ABCC2 c.-24C>T polymorphism increases the risk of resistance to antiepileptic drugs: a meta-analysis. J Clin Neurosci (2017) 37:6–14. 10.1016/j.jocn.2016.10.014 27816260

[156] Chen P Yan Q Xu H Lu A Zhao P. The effects of ABCC2 G1249A polymorphism on the risk of resistance to antiepileptic drugs: a meta-analysis of the literature. Genet Test Mol Biomarkers (2014) 18:106–11. 10.1089/gtmb.2013.0362 24325761 PMC3926164

[157] Wang Y Tang L Pan J Li J Zhang Q Chen B. The recessive model of MRP2 G1249A polymorphism decrease the risk of drug-resistant in Asian Epilepsy: a systematic review and meta-analysis. Epilepsy Res (2015) 112:56–63. 10.1016/j.eplepsyres.2015.02.007 25847339

[158] Palmeira A Sousa E Vasconcelos MH Pinto MM. Three decades of P-gp inhibitors: skimming through several generations and scaffolds. Curr Med Chem (2012) 19:1946–2025. 10.2174/092986712800167392 22257057

[159] Munagala S Sirasani G Kokkonda P Phadke M Krynetskaia N Lu P Synthesis and evaluation of *Strychnos* alkaloids as MDR reversal agents for cancer cell eradication. Bioorg Med Chem (2014) 22:1148–55. 10.1016/j.bmc.2013.12.022 24405813

[160] Summers MA Moore JL McAuley JW. Use of verapamil as a potential P-glycoprotein inhibitor in a patient with refractory epilepsy. Ann Pharmacother (2004) 38:1631–4. 10.1345/aph.1E068 15328394

[161] Iannetti P Spalice A Parisi P. Calcium-channel blocker verapamil administration in prolonged and refractory status epilepticus. Epilepsia (2005) 46:967–9. 10.1111/j.1528-1167.2005.59204.x 15946342

[162] Pirker S Baumgartner C Termination of refractory focal status epilepticus by the P-glycoprotein inhibitor verapamil. Eur J Neurol (2011) 18:e151 10.1111/j.1468-1331.2011.03513.x 22097953

[163] Asadi-Pooya AA Razavizadegan SM Abdi-Ardekani A Sperling MR. Adjunctive use of verapamil in patients with refractory temporal lobe epilepsy: a pilot study. Epilepsy Behav (2013) 29:150–4. 10.1016/j.yebeh.2013.07.006 23973639

[164] Borlot F Wither RG Ali A Wu N Verocai F Andrade DM. A pilot double-blind trial using verapamil as adjuvant therapy for refractory seizures. Epilepsy Res (2014) 108:1642–51. 10.1016/j.eplepsyres.2014.08.009 25223728

[165] Narayanan J Frech R Walters S Patel V Frigerio R Maraganore DM. Low dose verapamil as an adjunct therapy for medically refractory epilepsy – an open label pilot study. Epilepsy Res (2016) 126:197–200. 10.1016/j.eplepsyres.2016.07.004 27513375

[166] Loscher W Langer O. Imaging of P-glycoprotein function and expression to elucidate mechanisms of pharmacoresistance in epilepsy. Curr Top Med Chem (2010) 10:1785–91. 10.2174/156802610792928095 20645916 PMC3689923

[167] Cascorbi I ABC transporters in drug-refractory epilepsy: limited clinical significance of pharmacogenetics? Clin Pharmacol Ther (2010) 87:15–8. 10.1038/clpt.2009.237 20019695

[168] Bauer M Karch R Zeitlinger M Liu J Koepp MJ Asselin MC In vivo P-glycoprotein function before and after epilepsy surgery. Neurology (2014) 83:1326–31. 10.1212/WNL.0000000000000858 25186858 PMC4189097

[169] Engel J Wiebe S French J Sperling M Williamson P Spencer D Practice parameter: temporal lobe and localized neocortical resections for epilepsy: report of the Quality Standards Subcommittee of the American Academy of Neurology, in Association with the American Epilepsy Society and the American Association of Neurological Surgeons. Neurology (2003) 60:538–47. 10.1212/01.WNL.0000055086.35806.2D 12601090

[170] Morris GL III Gloss D Buchhalter J Mack KJ Nickels K Harden C Evidence-based guideline update: vagus nerve stimulation for the treatment of epilepsy: report of the Guideline Development Subcommittee of the American Academy of Neurology. Neurology (2013) 81:1453–9. 10.1212/WNL.0b013e3182a393d1 23986299 PMC3806910

[171] Perucca E French J Bialer M. Development of new antiepileptic drugs: challenges, incentives, and recent advances. Lancet Neurol (2007) 6:793–804. 10.1016/S1474-4422(07)70215-6 17706563

[172] Sorensen AT Kokaia M. Novel approaches to epilepsy treatment. Epilepsia (2013) 54:1–10. 10.1111/epi.12000 23106744

[173] Hocquemiller M Giersch L Audrain M Parker S Cartier N. Adeno-associated virus-based gene therapy for CNS diseases. Hum Gene Ther (2016) 27:478–96. 10.1089/hum.2016.087 27267688 PMC4960479

